# SPO11-Independent DNA Repair Foci and Their Role in Meiotic Silencing

**DOI:** 10.1371/journal.pgen.1003538

**Published:** 2013-06-06

**Authors:** Fabrizia Carofiglio, Akiko Inagaki, Sandra de Vries, Evelyne Wassenaar, Sam Schoenmakers, Christie Vermeulen, Wiggert A. van Cappellen, Esther Sleddens-Linkels, J. Anton Grootegoed, Hein P. J. te Riele, Bernard de Massy, Willy M. Baarends

**Affiliations:** 1 Department of Reproduction and Development, Erasmus MC - University Medical Center, Rotterdam, The Netherlands; 2 Division of Molecular Biology, Netherlands Cancer Institute, Amsterdam, The Netherlands; 3 Department of Obstetrics and Gynaecology, Erasmus MC - University Medical Center, Rotterdam, The Netherlands; 4 Erasmus Optical Imaging Centre, Department of Pathology, Erasmus MC - University Medical Center, Rotterdam, The Netherlands; 5 Institut de Génétique Humaine, CNRS UPR 1142, Montpellier, France; National Cancer Institute, United States of America

## Abstract

In mammalian meiotic prophase, the initial steps in repair of SPO11-induced DNA double-strand breaks (DSBs) are required to obtain stable homologous chromosome pairing and synapsis. The X and Y chromosomes pair and synapse only in the short pseudo-autosomal regions. The rest of the chromatin of the sex chromosomes remain unsynapsed, contains persistent meiotic DSBs, and the whole so-called XY body undergoes meiotic sex chromosome inactivation (MSCI). A more general mechanism, named meiotic silencing of unsynapsed chromatin (MSUC), is activated when autosomes fail to synapse. In the absence of SPO11, many chromosomal regions remain unsynapsed, but MSUC takes place only on part of the unsynapsed chromatin. We asked if spontaneous DSBs occur in meiocytes that lack a functional SPO11 protein, and if these might be involved in targeting the MSUC response to part of the unsynapsed chromatin. We generated mice carrying a point mutation that disrupts the predicted catalytic site of SPO11 (*Spo11^YF/YF^*), and blocks its DSB-inducing activity. Interestingly, we observed foci of proteins involved in the processing of DNA damage, such as RAD51, DMC1, and RPA, both in *Spo11^YF/YF^* and *Spo11* knockout meiocytes. These foci preferentially localized to the areas that undergo MSUC and form the so-called pseudo XY body. In SPO11-deficient oocytes, the number of repair foci increased during oocyte development, indicating the induction of S phase-independent, *de novo* DNA damage. In wild type pachytene oocytes we observed meiotic silencing in two types of pseudo XY bodies, one type containing DMC1 and RAD51 foci on unsynapsed axes, and another type containing only RAD51 foci, mainly on synapsed axes. Taken together, our results indicate that in addition to asynapsis, persistent SPO11-induced DSBs are important for the initiation of MSCI and MSUC, and that SPO11-independent DNA repair foci contribute to the MSUC response in oocytes.

## Author Summary

Meiosis is a special cell division that generates genetically divergent haploid germ cells. At the very beginning of this process, during meiotic prophase, the enzyme SPO11 generates hundreds of DNA double-strand breaks (DSBs). Meiotic DSBs are repaired via a mechanism that requires the presence of an intact homologous template. This repair process stimulates homologous chromosome pairing, and the formation of a protein complex that connects the paired chromosome axes, reaching a state called synapsis. Male mammals carry a pair of largely heterologous sex chromosomes, the X and Y, which show delayed DSB repair and extensive asynapsis. In addition, the X and Y chromosomes are transcriptionally silenced by a mechanism named Meiotic Sex Chromosome Inactivation (MSCI). This mechanism is a specialization of a more general silencing mechanism, named Meiotic Silencing of Unsynapsed Chromatin (MSUC), that is induced when any pairing problem between homologous chromosomes results in asynapsis, in male as well as female meiotic prophase cells. Here, we demonstrate that in addition to asynapsis, the persistent presence of DNA repair foci is a hallmark of meiotic silencing. In addition, we show that SPO11-independent DNA repair foci form during normal oocyte development. We propose that these foci represent sites of unrepaired DSBs that are capable of inducing transcriptional silencing, irrespective of synapsis.

## Introduction

During meiotic prophase in yeast and mammals, the induction of DNA double-strand breaks (DSBs) by the transesterase SPO11 precedes stable pairing and synapsis of homologous chromosomes [1], [2]. Synapsis between chromosomes is achieved by the formation of a specific protein complex, consisting of lateral elements along the chromosomal axes that contain SYCP2, SYCP3 [3], [4], different components of the cohesin complex [5], [6], and (before synapsis is achieved, on axial elements) the HORMA-domain proteins HORMAD1 and HORMAD2 [7], [8]. Lateral elements are connected by a central element containing SYCP1 [9] and several other meiosis-specific proteins, including SYCE1, SYCE2 [10] and TEX12 [11]; reviewed by Yang and Wang [12]. Parallel to synaptonemal complex formation, meiotic DSBs are repaired, thereby facilitating homologous chromosomes interaction and the achievement of complete synapsis.

In male mammals, the X and Y chromosomes form a very special pair; they can synapse only in their short pseudoautosomal regions, and localize to the periphery of the nucleus. Furthermore, the XY chromatin is silenced, forming the XY body, by a process named meiotic sex chromosome inactivation (MSCI). This requires the expression of the histone variant H2AX [13]. The checkpoint kinase ATR phosphorylates H2AX at S139, generating γH2AX [14]. γH2AX is the earliest known marker of MSCI. This specific histone modification is also found in somatic cells, usually at sites of DNA DSB repair [15]. Interestingly, H2AX phosphorylation in response to DNA damage has been coupled to reduced levels of RNA polymerase II activity in somatic cells [16].

MSCI is considered a specialized form of a more general mechanism termed meiotic silencing of unsynapsed chromatin (MSUC), which silences unsynapsed chromatin in male and female meiotic prophase cells [17]–[19]. The exact cascade of events that leads to this transcriptional silencing is not known, but it has been established that there is a tight correlation between the presence of unsynapsed chromosomal axes coated by HORMAD1 and HORMAD2 (the two mammalian orthologs, of the yeast protein Hop1 [7], [20], [21]), the accumulation of ATR along these axes, the formation of γH2AX, and the transcriptional silencing. Indeed, it was recently reported that efficient accumulation of ATR on the XY body requires the HORMAD1 and HORMAD2 proteins [22], [23]. Many DNA repair proteins accumulate at the XY body, together with histone modifications such as specific methylation, sumoylation and ubiquitylation (reviewed by Inagaki et al. [24]). The accumulation of DSB repair proteins may be caused by delayed or stalled DSB repair in regions that fail to synapse. Persistent meiotic DSBs can indeed be observed on the X, but not on the Y chromosome, via immunocytochemical detection of the homologous recombination proteins RAD51 and its meiosis-specific paralogue DMC1 [25]–[28]. RAD51 and DMC1 have DNA-dependent ATPase activity and form filaments on single-stranded resected DNA-ends at DSB repair sites, and are essential for homologous recombination repair in mitotic and meiotic cells, respectively [29]–[32].

Evidence for a relationship between meiotic DSBs and homologous synapsis is provided by the observation that synapsis is severely affected in the absence of SPO11-induced meiotic DSBs [33], [34]. Some heterologous synapsis can occur in *Spo11* knockout meiocytes, but both spermatocytes and oocytes do not proceed beyond a zygotene-like stage [33], [34]. In *Spo11* knockout spermatocytes, a pseudo XY body is formed, which most often does not localize to the X and Y chromosomes, but to part of the unsynapsed chromatin [35], [36]. It has been defined as a condensed chromatin structure that, similar to the XY body, is marked by γH2AX and ATR, and is transcriptionally silenced [35], [37]. Based upon these characteristics, it has been proposed that the pseudo XY body is a manifestation of MSUC [37]. However, in *Spo11* knockout spermatocytes, HORMAD1 and HORMAD2 coat all unsynapsed axes, but the pseudo XY body forms only on part of the unsynapsed chromatin, indicating that somehow the MSUC response is not complete [7], [8] In addition, although more than 60% of the spermatocyte nuclei in *Spo11* knockout testes contain a pseudo XY body, only 11% show clear accumulation of ATR along the unsynapsed axes in the pseudo XY body, compared to 100% ATR accumulation along the axes of true XY bodies in wild type spermatocytes [23]. The restriction of MSUC to only part of the unsynapsed chromatin is surprising, and raises the possibility that, apart from asynapsis, also other mechanisms may contribute to the activation of MSUC and MSCI. Since all known players in these processes function also in DNA repair we hypothesized that persistent DSBs on unsynapsed axes may contribute to the activation of MSUC and MSCI. This would then suggest that, even in the absence of SPO11, perhaps some damage-induced DSBs are frequently present, and could play a role in restricting the MSUC response to those areas that contain both unsynapsed axes and DNA damage. This notion is supported by the fact that radiation-induced DSBs in mouse leptotene cells enhance the efficiency of MSUC of a small translocation bivalent that carries a heterologous region of approximately 35–40 Mb [38]. In addition, recent data also provide a link between DSB repair, the checkpoint kinase ATM, and transcriptional silencing of surrounding chromatin in somatic cells [39].

Herein, we have generated a mouse model with a point mutation, which inactivates the catalytical site of SPO11. We used this mouse model to obtain more insight in the relation between the presence of DSBs and MSUC.

As expected based on our hypothesis, we found that SPO11-independent DNA repair foci are present in spermatocytes and oocytes. Moreover, we observed *de novo* induction of DNA repair foci in zygotene-like SPO11-deficient oocytes. Together with the results of a thorough analysis of the relationship between the localisation of DSB repair proteins and the MSUC response, our data reveal a direct link between the presence of persistent damage and the activation of MSUC and MSCI.

## Results

### Generation and initial analysis of the Spo11 Y138F mutation

**Figure 1:**
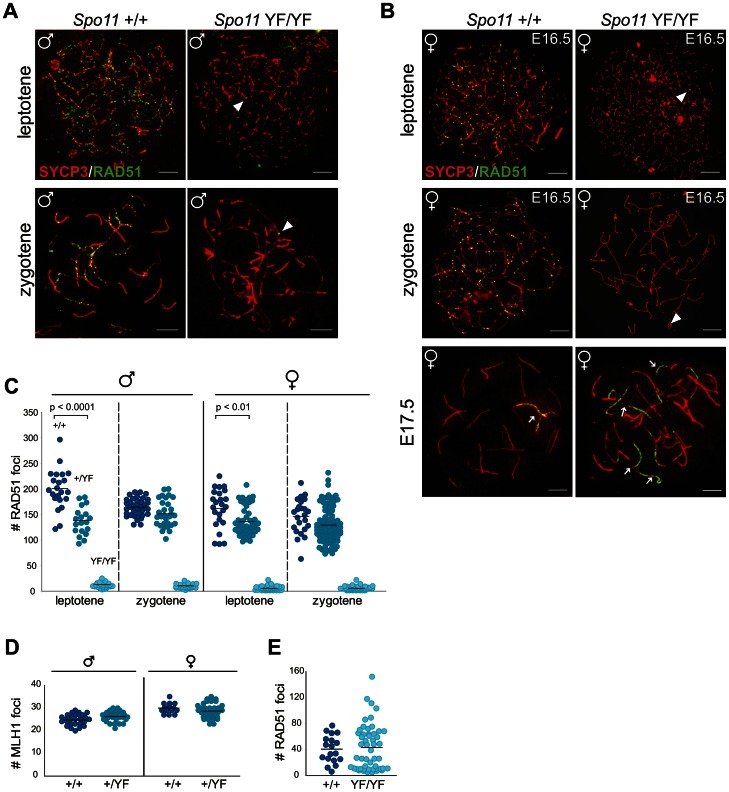
SPO11-dependent and -independent RAD51 foci in mouse meiocytes. (A–C) The number of RAD51 foci decreases from leptotene to zygotene in *Spo11^+/+^* and *Spo11^+/YF^* spermatocytes, whereas a few foci are detected in *Spo11^YF/YF^* spermatocytes and oocytes at both stages. (A–B) Double immunostaining with anti-SYCP3 (red), anti-RAD51 (green) of spermatocyte (A) and oocyte (B) nuclei from *Spo11^+/+^* (A–B, left panel) and *Spo11^YF/YF^* (A–B, right panel) mice. Arrowheads point to RAD51 foci in *Spo11^YF/YF^* spermatocytes and oocytes, both leptotene and zygotene. Extensive accumulation of RAD51 along axial elements of one or few chromosomes (arrows) can be observed in both *Spo11^+/+^* and *Spo11^YF/YF^* oocyte nuclei (B, lower panel). Size bars represent 10 µm. (C) The number of RAD51 foci was counted in *Spo11^+/+^*, *Spo11^+/YF^*, and *Spo11^YF/YF^* leptotene and zygotene spermatocytes and oocytes. Each dot represents the focus count of one nucleus. Black bars indicate mean number of foci. P values for the indicated comparisons (Mann-Whitney, two-tailed), and genotypes are indicated in the plot. (D) The number of MLH1 foci in pachytene spermatocyte nuclei was counted in *Spo11^+/+^*and *Spo11^+/YF^* mice. Black bars indicate the mean values. (E) Number of RAD51 foci at E17.5 in *Spo11^+/+^* and *Spo11^YF/YF^* oocytes.

We used a *Spo11* knock-in mouse model in which the catalytically active tyrosine (Tyr) 138 residue is replaced by a phenylalanine (Phe) (*Spo11^YF/YF^*) (Figure S1A, B). In yeast and plants, mutation of the analogous Tyr residue abolished meiotic DSB formation [40]–[42], and a similar mouse mutant was recently described [43]. Presence of the point mutation and normal expression of the mutant protein were verified by sequence analyses, RT-PCR, and Western blot analyses (Figure S1C, D, E). The amount of mutant and/or wild type SPO11 protein in the testis of +/+, +/YF and YF/YF animals was comparable. Identical to the *Spo11* knockout [33], [34], male and female *Spo11^YF/YF^* mice are infertile, and leptotene and zygotene nuclei display global absence of markers of DSB formation and repair (Figure 1A, B, and C). Spermatocytes and oocytes reach a zygotene-like stage with variable degrees of heterologous synapsis (Figure S2A, B, C).

### A two-fold reduction in the amount of functional SPO11 reduces the number of RAD51 foci at leptotene but not at zygotene

We analyzed the formation of meiotic DSBs in wild type, heterozygote and homozygote *Spo11^YF/YF^* mice through immunocytochemical analysis of the formation of RAD51 foci. The number of RAD51 foci was quantified in leptotene and zygotene spermatocyte and oocyte nuclei (Figure 1). In wild type leptotene, many DSBs are formed, concomitant with the assembly of short patches of axial element along the chromosomal axes (Figure 1A, B, left panels, 1C). In zygotene, repair of meiotic DSBs occurs, parallel to the pairing of homologous chromosomes. Axial elements of paired homologous chromosomes then synapse (and are therefore termed lateral elements), through the formation of the central element of the synaptonemal complex (SC) (Figure 1A, B, left panel). The number of RAD51 foci gradually decreases, from leptotene to zygotene (Figure 1 A, C), as has been observed before [26]. It should be noted that, in mouse, male meiosis induction occurs throughout postpubertal life, whereas female meiosis is initiated only once during embryonic development (around embryonic day 13 (E13)). Oocytes progress through leptotene and zygotene in 15–20 h [44], [45]. At E17, the vast majority of oocytes has reached the pachytene stage, and around E19, oocytes enter diplotene, reaching the first meiotic arrest. Spermatocytes require a longer time span between leptotene (induction of DSBs) and early pachytene (synapsis) of approximately 48 h [46]. In *Spo11^+/YF^* leptotene spermatocyte nuclei, the number of RAD51 foci was approximately 30% lower compared to wild type (Figure 1C). However, in zygotene nuclei, no difference in the number of RAD51 foci between wild type and heterozygote nuclei was observed (Figure 1C). Similar to the males, the number of RAD51 foci was lower in *Spo11^+/YF^* leptotene oocytes, compared to the wild type, and a small difference between the wild type and heterozygote oocytes was still observed at zygotene (Figure 1C). MLH1 is mismatch repair protein that is a well-known marker of crossover sites [47], and functions in the resolution of joint molecules at the final phase of crossover formation [48]. The number of MLH1 foci was not different between wild type and *Spo11^+/YF^* spermatocytes (Figure 1D).

### SPO11-independent DNA repair foci in *Spo11^YF/YF^* and *Spo11^−/−^* meiocytes

In *Spo11^YF/YF^* animals, a few RAD51 foci were observed on the axial elements in leptotene and zygotene-like spermatocytes (average foci number 12±4.4, n = 54) and oocytes (average foci number 5±3.7, n = 50) (Figure 1A–C). Surprisingly, from E17.5 onwards, when oocytes should have reached the pachytene stage, we observed *de novo* RAD51 accumulation (Figure 1E), in oocytes from *Spo11^YF/YF^* mice. These RAD51 foci formed along most of the length of one or more axes (Figure 1B, lower panel, right). Such marked accumulation of RAD51 is also observed in wild type and *Spo11^+/YF^* pachytene oocytes (Figure 1B, lower panel, left), but in a relatively small proportion of the nuclei (around 20%, see also below). To confirm the specificity of this pattern of RAD51 accumulation, we also used a commercial RAD51 antibody previously reported to mark RAD51 foci in spread meiotic nuclei [49]. This antibody yielded a similar pattern of RAD51 accumulation in oocytes (Compare Figure 1B to Figure S3). To ensure that the RAD51 foci that are observed in *Spo11^YF/YF^* spermatocytes and oocytes are not caused by remnant SPO11 activity, we also analysed RAD51 localisation in *Spo11* knockout meiocytes. As expected, the pattern of RAD51 foci staining in *Spo11* knockout spermatocytes and oocytes was similar to what was observed in meiocytes of *Spo11^YF/YF^* animals (Figure S4). This confirms that the observed RAD51 foci in our *Spo11^YF/YF^* model are SPO11-independent.

### A pseudo XY body is present in *Spo11^YF/YF^* spermatocytes, and in *Spo11^+/+^* and *Spo11^YF/YF^* oocytes

Extensive asynapsis is thought to elicit an MSUC response, which can be observed in *Spo11^−/−^* spermatocytes as a γH2AX positive domain in the nucleus [36], [37]. This domain has been termed pseudo XY body, since it does not necessarily include chromatin from the X and Y chromosomes.

Similar to what has been described for *Spo11* knockout mice, we observed one or two pseudo XY bodies in late zygotene-like spermatocytes from *Spo11^YF/YF^* mice (Figure S5A). In addition to γH2AX, other components of the DNA repair machinery are known to accumulate on the unsynapsed axes of the pseudo XY body (BRCA1, TOPBP1), or on the surrounding chromatin (MDC1) in *Spo11* knockout spermatocytes [37], [50], and this was also observed for the pseudo XY bodies in *Spo11^YF/YF^* spermatocytes (Figure S5B–D).

As recently reported, pseudo XY body-like structures can also be detected in *Spo11* knockout oocytes [22], and even wild type oocytes have been reported to contain a MSUC region in a small percentage of the pachytene oocytes that fails to correctly synapse all chromosomes [51]. We also observed areas of MSUC in a minority of wild type and *Spo11^+/YF^*oocytes at E16.5 and E17.5 (Table 1). In addition, in *Spo11^YF/YF^* ovaries we observed a γH2AX-positive chromatin domain in about 14% of oocytes at E16.5 (Table 2), and in more than 80% of oocytes derived from *Spo11^YF/YF^* ovaries at E17.5 (Table 2).

**Table 1:** Number of different subtypes of meiotic nuclei and frequency of pachytene nuclei with a pseudo-XY body in E16.5 and E17.5 oocytes from *Spo11^+/+^* and *Spo11^+/YF^*embryos.

	genotype	# leptotene (%)*	# zygotene (%)*	# pachytene (%)*		fraction of pachytenes with pseudo XY body
				pseudo XY body (+)	pseudo XY body (−)	
**E16.5**	***Spo11*** ^+/+^	13 (8.4)	77 (50)	10 (6.5)	54 (35)	0.16
	***Spo11*** ^+/***YF***^	10 (6.5)	105 (68)	7 (4.5)	32 (21)	0.18
**E17.5**	***Spo11*** ^+/+^	0 (0)	6 (4.3)	32 (23)	103 (73)	0.24
	***Spo11*** ^+/***YF***^	0 (0)	6 (3.8)	34 (22)	118 (75)	0.23

* **percentage of the total number of counted nuclei.**

**Table 2:** Number of different subtypes of meiotic nuclei and frequency of nuclei with a pseudo-XY body in E16.5 and E17.5 oocytes from *Spo11^YF/YF^*embryos.

	genotype	# leptotene (%)*	# zygotene (%)*	
			pseudo XY body (+)	pseudo XY body (−)
**E16.5**	***Spo11^YF/YF^***	33 (26)	18 (14.2)	76 (59.8)
**E17.5**	***Spo11^YF/YF^***	0 (0)	99 (81.1)	23 (18.9)

* **percentage of the total number of counted nuclei.**

**Figure 2:**
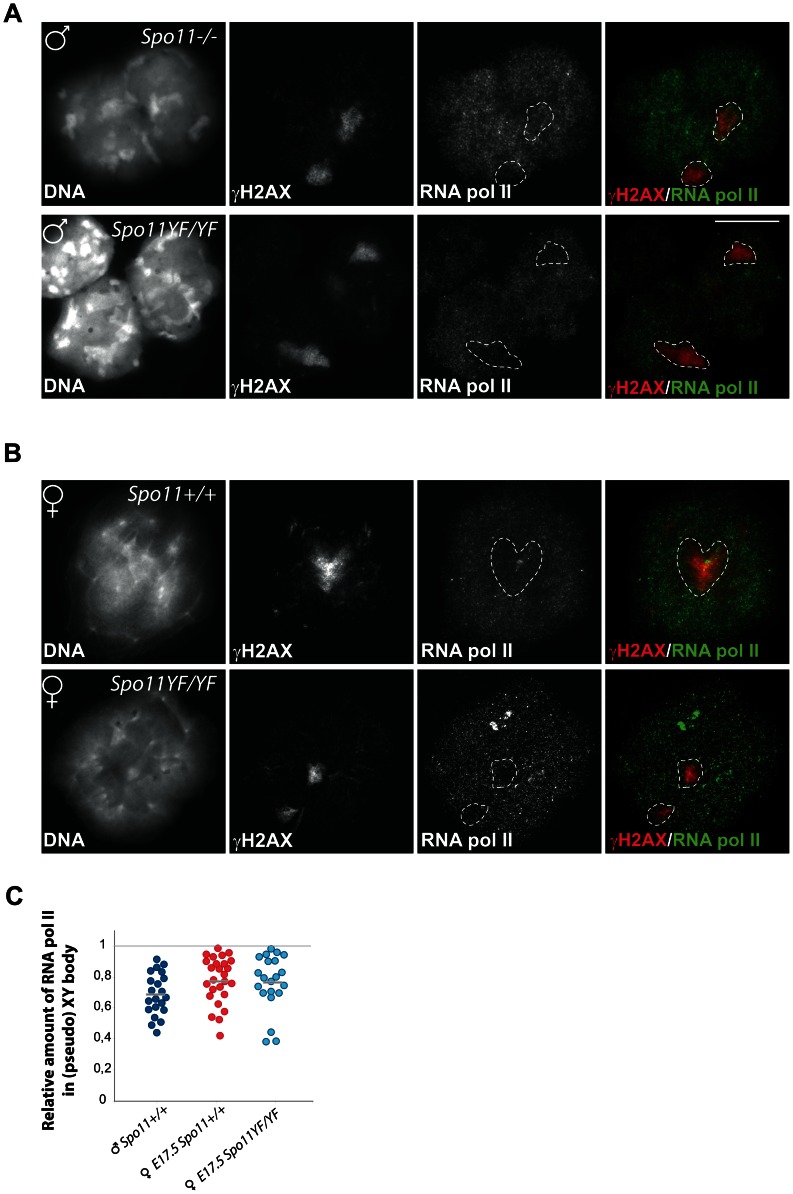
Transcriptional silencing of the pseudo XY body in spermatocytes and oocytes. (A–B) Double immunostaining with anti-γH2AX and anti-RNA polymerase II of spermatocyte (A) and oocyte (B) nuclei from *Spo11^−/−^* (A, upper panel), *Spo11^YF/YF^* (A–B, lower panels), and *Spo11^+/+^* (B, upper panel) animals. Nuclear domains enriched in γH2AX are marked by a dashed circle. (C) Scatter plots of the relative amount of RNA polII in a γH2AX domain normalized to the RNA polII level in a non-heterochromatic area of the same nucleus. Every dot represents a nucleus. RNA polII levels are compared between γH2AX domains (pseudo XY body) of *Spo11^+/+^* and *Spo11^YF/YF^* E17.5 oocytes, and the proper sex body in *Spo11^+/+^* mid-pachytene spermatocytes. Grey lines indicate the average. No significant difference between the wild type pachytene spermatocyte nuclei and either E17.5 oocyte nuclei group was observed (Mann-Whitney, confidence interval p<0.01).

The transcriptional silencing in the XY body can be immunocytochemically visualized as an area that is relatively depleted of RNA polymerase II [17]. To verify that the γH2AX domain detected in SPO11-deficient spermatocytes and oocytes is a transcriptionally silenced region, we performed RNA polymerase II (RNA pol II) staining and indeed observed a depletion of this enzyme from the areas enriched for γH2AX in *Spo11^−/−^* and *Spo11^YF/YF^*spermatocytes and oocytes (Figure 2A and B). To verify the results, we quantified the relative average intensity of RNA pol II staining in the γH2AX domain in oocytes, and compared it to the relative intensity in the true XY body of wild type pachytene spermatocytes (Figure 2C). Despite the fact that we observed variable depletion levels within each of the three analysed categories, the relative average level of RNA pol II in γH2AX domains of wild type (0.77±0.16, n = 30) and *Spo11^YF/YF^* (0.76±0.18, n = 30) oocytes is similar, and also comparable to what is observed for the XY body in male wild type spermatocytes (0.69±0.14, n = 30) (Mann-Whitney, confidence interval p<0.001), indicating a significant transcriptional silencing.

Based on these results, we will refer to the γH2AX domains that are observed in both *Spo11^YF/YF^* and *Spo11^+/+^* oocytes as pseudo XY bodies.

### RAD51 foci frequently localize to the pseudo XY body in *Spo11^YF/YF^* spermatocytes

Having established that both SPO11-independent DNA repair foci and pseudo XY bodies are present in SPO11-deficient spermatocytes and oocytes, we subsequently analysed whether these foci are indeed associated with the MSUC areas. Such an association would be expected, if SPO11-independent DNA damage, present on part of the unsynapsed axes, plays a role in nucleating the formation of the pseudo XY body. To investigate this, we performed co-immunostaining experiments for RAD51 to visualize DSB repair sites, γH2AX to visualize the pseudo XY body and SYCP3 to assess the stages of the cells.

Due to the severe impairment of meiotic prophase progression in *Spo11^YF/YF^* animals, spermatogenesis is arrested at stage IV, but spermatocytes never reach a true pachytene stage. We performed our analyses on a subpopulation of spermatocytes which displayed one or more areas of (heterologous) synapsis and showed no signs of SC fragmentation, in order to select healthy spermatocytes which had already entered the zygotene stage.

**Figure 3:**
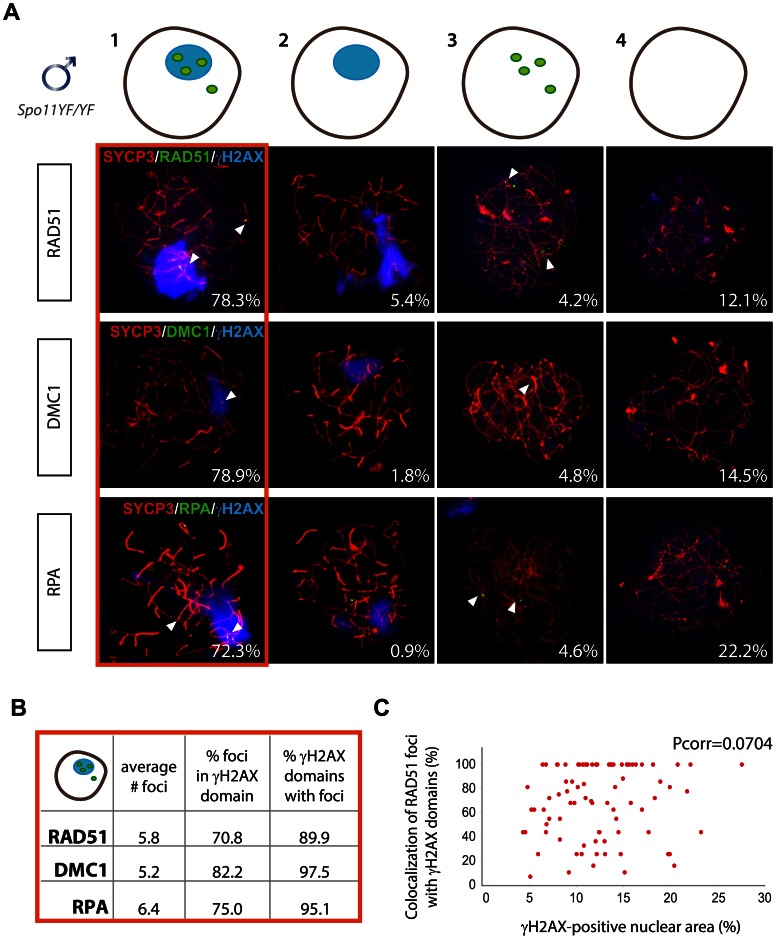
Enrichment of DNA repair markers in the pseudo XY body of *Spo11^YF/YF^* spermatocytes. (A) Nuclei of *Spo11^YF/YF^*zygotene spermatocytes were divided in four subgroups depending on their positivity for the pseudo XY body and for foci of one of the three DNA repair proteins RAD51 (n = 120), DMC1 (n = 227) or RPA (n = 108) as follows: 1) with pseudo XY body and with foci, 2) with pseudo XY body and without foci, 3) without pseudo XY body and with foci, 4) without pseudo XY body and without foci. Spermatocyte nuclei were immunostained with anti-SYCP3 (red), anti-γH2AX (blue), and one of the following antibodies: anti-RAD51 (green, upper panel), anti-DMC1 (green, middle panel) or RPA (green, lower panel). Every panel shows a representative nucleus for each of the four subgroups mentioned above. Numbers in the bottom left corner of every picture represent the percentage of nuclei of this type in the analyzed cell population. (B) The average number of RAD51, DMC1 and RPA foci per nucleus was counted in spermatocytes of the first subgroup (outlined in red). The table also shows the percentage of foci located within a pseudo XY body and the percentage of pseudo XY bodies which contained at least one focus. (C) Scatter plot representing the colocalization percentage in relation to the fraction of the nuclear area occupied by the pseudo XY body. Every dot represents a nucleus. Pearson linear correlation coefficient [Pcorr] = 0.0741.

First of all we determined the frequency of spermatocytes with RAD51 foci and with a pseudo XY body. We split our population (n = 240) in four classes (Figure 3A): 1) cells having both a pseudo XY body and RAD51 foci; 2) cells having only a pseudo XY body; 3) cells having only RAD51 foci; and 4) cells lacking both a pseudo XY body and RAD51 foci (Figure 3A, B). The results indicate that the vast majority of nuclei (78.3%) contain both a pseudo XY body as well as RAD51 foci. Although RAD51 is a well-known marker of sites of DSB repair [52], it may also accumulate on ssDNA that is formed in a different context of DNA damage, such as observed during collapse of a replication fork in S phase [53]. To obtain additional evidence for the presence of DNA damage in *Spo11^YF/YF^* spermatocytes, we performed the same analysis by staining for two more markers of DNA damage and repair: DMC1 and RPA. DMC1 is the meiosis-specific homolog of RAD51 which participates in the process of repair of meiotic DSBs via homologous recombination. Hence, we expected the results for DMC1 and RAD51 to be similar. Indeed, comparable percentages of the analyzed nuclei were found to fall in each of the four classes (Figure 3A). In addition, we observed colocalization between RAD51 and DMC1 foci in the γH2AX domains (Figure S6A).

Unlike RAD51 and DMC1, RPA is not a recombinase but a single-stranded DNA (ssDNA) binding protein which takes part in many processes involving DNA metabolism (reviewed by Sakaguchi et al. [54]). At meiotic DSBs, the dynamics of RPA foci differ from those of DMC1, and although both proteins are enriched on the XY body, this occurs at different developmental time points (Figure S7). Nevertheless, similar to what was found for RAD51 and DMC1, 72.3% of the cells (n = 108) showed presence of both RPA foci and γH2AX domains (Figure 3A, lower panel).

The high percentages of cells with a pseudo XY body and DNA damage markers, provided an indication for a possible correlation between the presence of DNA damage, in particular DSBs, and the formation of the pseudo XY body. To further test the hypothesis for such a correlation, we determined the colocalization between each DNA repair marker and the γH2AX domain, in the fraction of spermatocytes that was positive for both of these features. We counted similar average numbers of RAD51, DMC1 and RPA foci (5.7, 5.2 and 6.4, respectively) in the nuclei, and the percentages of colocalization with the γH2AX domain(s) ranged between 70.8% (RAD51) and 82.2% (DMC1) (Figure 3B). Furthermore, up to 89–98% of the analysed pseudo XY bodies contained at least one focus of RAD51, DMC1 or RPA (Figure 3B).

To validate that the frequent localization of RAD51 in the pseudo XY body is not coincidental, we compared the relative area of the nucleus that was positive for γH2AX (pseudo XY body) to the fraction of RAD51 foci that was found inside that area. We observed that the fraction of RAD51 that localized inside the pseudo XY body (more than 70%) was much larger than the fraction of the nucleus that was taken up by this chromatin domain (20% of the total area). In addition, there was no specific correlation (Pearson linear correlation coefficient [Pcorr] = 0.0704) between the size of the pseudo XY body and the percentage of RAD51 foci that was found in the pseudo XY body (Figure 3C). In *Spo11* knockout spermatocytes, a similar pattern of colocalization between RAD51, DMC1, and RPA foci and the pseudo XY body was observed (Figure S8).

### Radiation induced DSBs elicit an MSUC response in *Spo11^YF/YF^* spermatocytes

**Figure 4:**
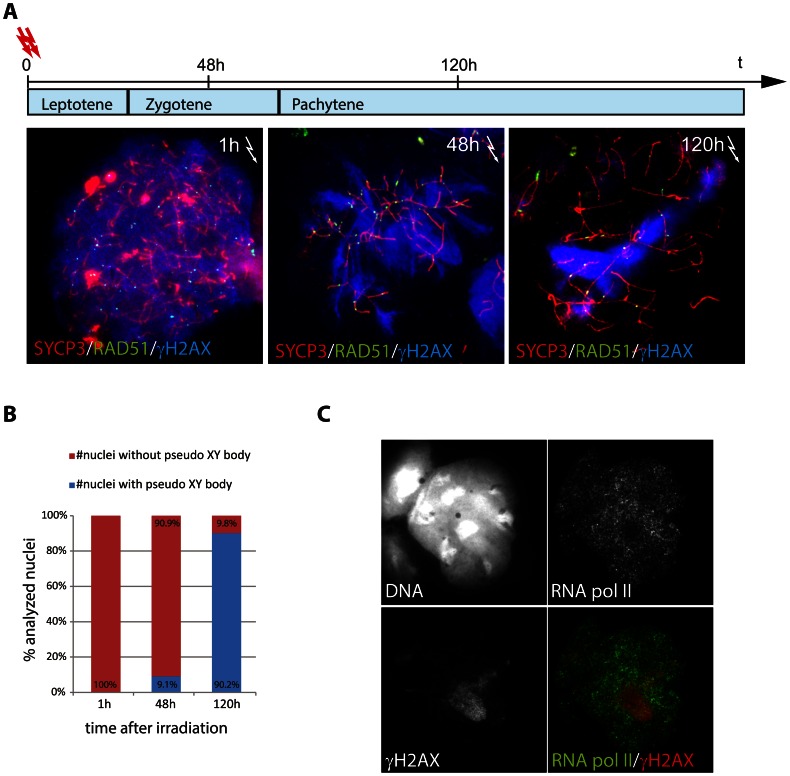
Relocalisation of persistent radiation-induced DSBs into a pseudo XY body. (A) Irradiated *Spo11^YF/YF^* spermatocytes were collected 1 h, 48 h and 120 h upon irradiation and immunostained for RAD51 (green), SYCP3 (red), and γH2AX (blue). Spermatocytes that were irradiated at the leptotene stage, should have reached zygotene and pachytene with respect to the pattern of γH2AX, at 48 and 120 h following irradiation, respectively. (B) Fraction of cells showing a pseudo XY body upon irradiation at the analysed time-points (n = 50). (C) Immunostaining of *Spo11^YF/YF^* spermatocyte 120 hours after irradiation with anti-RNA pol II (green) and anti-γH2AX (red). The intense γH2AX domain (pseudo XY body) corresponds to a nuclear area depleted for RNA pol II.

The localised presence of DNA repair foci in one or a few pseudo XY bodies indicates that DNA damage in spermatocytes tends to concentrate in a single, transcriptionally silenced area. To test this hypothesis, we induced exogenous DSBs in *Spo11^YF/YF^* spermatocyte nuclei by whole-body irradiation, and analysed the presence of DSB markers at different time points following the treatment. We observed approximately 120 (±5.3, n = 30) RAD51 foci and a nucleus-wide accumulation of γH2AX at 1 h following irradiation. Interestingly, 48 hours after irradiation, we still observed extensive H2AX phosphorylation emanating from the many RAD51 foci (Figure 4A). However, 120 h following irradiation, when cells that were irradiated at leptotene would have progressed to pachytene in a wild type background, a pseudo XY body was observed in about 90% (n = 70) of the analysed nuclei (Figure 4B). These pseudo XY bodies always contained RAD51 foci (25.1±1.73, n = 50), and the majority of the radiation-induced RAD51 foci that are still present at this time point (65.7%) localized in the pseudo XY body (Figure 4A). These data show that the persistent radiation-induced DSBs tend to relocalize in a specific nuclear subdomain. This phenomenon is in accordance with the colocalization of unsynapsed or partially synapsed translocation chromosomes, carrying persistent meiotic DSBs, with the XY body [38].

To confirm that the pseudo XY body in these irradiated spermatocytes is an MSUC area, as observed in non-irradiated *Spo11^YF/YF^* spermatocytes, we performed co-immunostaining for γH2AX and RNA pol II. We detected a depletion of this enzyme in the areas enriched for γH2AX, indicating that they are transcriptionally silenced (Figure 4C).

### Pseudo XY bodies in *Spo11^YF/YF^* oocytes correlate with DSB markers

Next, we asked if RAD51, DMC1, and RPA foci also preferentially localized in the pseudo XY bodies in E17.5 *Spo11^YF/YF^* oocytes.

**Figure 5:**
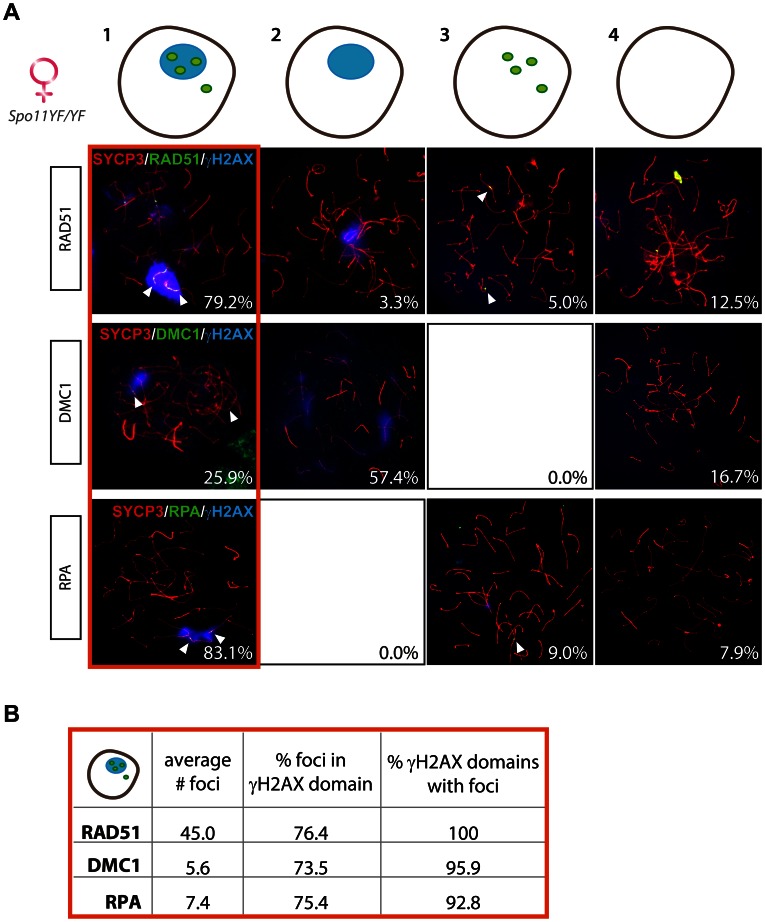
Enrichment of DNA repair markers in the pseudo XY body of *Spo11^YF/YF^* oocytes. (A) Oocyte nuclei from *Spo11^YF/YF^* E17.5 embryos were immunostained with anti-SYCP3 (red), anti-γH2AX (blue), and one of the following antibodies: anti-RAD51 (green, upper panel), anti-DMC1 (green, middle panel), or RPA (green, lower panel). Foci of each marker listed above are indicated with arrowheads. Quantification of the four fractions (defined in the legend to Figure 3A) was performed in 120, 54, and 89 oocyte nuclei, for RAD51, DMC1, and RPA protein foci, respectively. Numbers in the bottom left corner of every picture represent the percentage of nuclei of this type in the analyzed cell population. (B) The number of RAD51, DMC1 and RPA foci was counted in oocyte nuclei showing both a pseudo XY body and foci (red circle). The average total number of foci of each protein per nucleus is reported in the first column of the table. The second and the third column show the percentage of foci located within a pseudo XY body and the percentage of pseudo XY bodies that contained at least one focus.

As discussed above, RAD51 was found to accumulate extensively on some chromosomal axes, often coating them completely, so that single foci could not be easily resolved. Such marked accumulation was not observed for DMC1 or RPA, which are forming fewer foci (average number of 5.6±2.3, n = 20 and 7.4±6.9, n = 30, respectively). Despite this difference in foci pattern, the percentage of oocyte nuclei that contained both a γH2AX domain and RAD51 foci (79.2%, n = 120) was similar to the percentage of oocyte nuclei with a γH2AX domain and RPA foci (83.1%, n = 89) (Figure 5A, upper and lower panel respectively). In contrast, only 25.9% of the analysed *Spo11^YF/YF^* oocytes (n = 54) displayed DMC1 foci, but all these cells also had a γH2AX domain. The rest of the nuclei had only a pseudo XY body (57.41%) or were negative for both DMC1 and γH2AX (16.67%) (Figure 5A, middle panel).

In the group of nuclei that contained both RAD51 foci and a γH2AX domain, the pseudo XY body always contained RAD51 foci that coated part of the axes (Figure 5B). Also, in E17.5 *Spo11^YF/YF^* oocytes that contained a pseudo XY body and DMC1 or RPA foci, more than 90% of the pseudo XY bodies contained DMC1 or RPA foci, respectively. Conversely, the vast majority of RAD51, DMC1, and RPA foci in this subgroup of nuclei were located in the pseudo XY body, similar to what was observed for *Spo11^YF/YF^* spermatocyte nuclei. Furthermore, the DMC1 foci were found to colocalize with some of the (more abundant) RAD51 foci in the pseudo XY bodies of oocytes (Figure S6B).

For comparison, these analyses were also performed on *Spo11* knockout E17.5 oocytes and this provided similar results (Figure S8, right).

### DSB repair proteins mark pseudo XY bodies that are occasionally observed in wild type oocytes

Interestingly, also in wild type and *Spo11^YF/+^* oocyte nuclei, RAD51 coats the axial elements in γH2AX-positive domains (Table 1). These pseudo XY bodies were observed in approximately 20% of pachytene oocytes, similar to what was previously reported by Koutznetsova et al. [51] who observed BRCA1 and ATR on unsynapsed axes in around 15% of the oocyte population from E17 wild type embryos.

**Figure 6:**
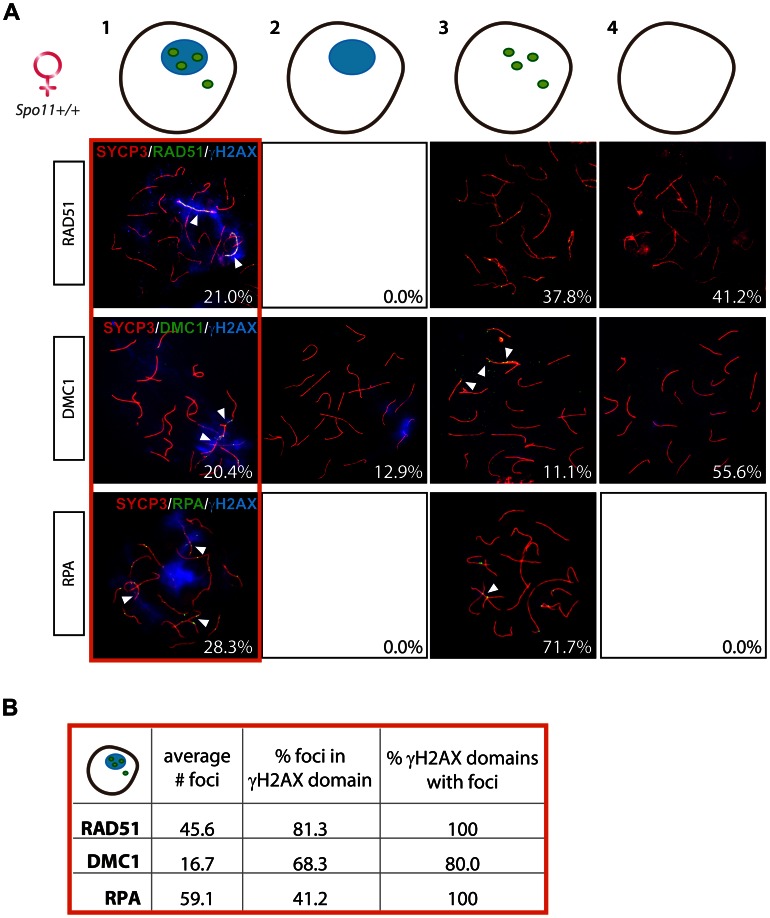
Enrichment of DNA repair markers in the pseudo XY bodies of *Spo11^+/+^* oocytes. (A) Oocyte nuclei from *Spo11^+/+^* E17.5 embryos were immunostained with anti-SYCP3 (red), anti-γH2AX (blue) and anti-RAD51 (green, upper panel) or anti-DMC1 (green, middle panel) or RPA (green, lower panel). Foci of each marker listed above are indicated with arrowheads. Numbers in the bottom left corner of every picture represent the percentage of nuclei of the respective type in the analyzed cell population. Quantification of the four fractions (defined in Figure 3A) was performed in 271, 54, and 53 oocyte nuclei, for RAD51, DMC1, and RPA protein foci, respectively. (B) The number of RAD51, DMC1, and RPA foci was counted in pachytene oocyte nuclei showing both a pseudo XY body and foci (red circle). The average total number of foci of each protein per nucleus is reported in the first column of the table. The second and the third column show the percentage of foci located within a pseudo XY body-like domain and the percentage of pseudo XY body-like domains which contained at least one focus.

To analyse this further, we studied the localisation of other proteins involved in homologous recombination (DMC1 and RPA) in relation to the formation of a γH2AX domain. Again we divided the oocyte population in four subgroups, based on the detection of γH2AX and the three DNA repair markers. As expected, the majority of pachytene oocytes showed complete synapsis of all chromosomes and no clear γH2AX-positive domain. Around 20–30% of nuclei showed pseudo XY bodies, as defined by the presence of one or a few distinct γH2AX-positive domains (Figure 6A). Approximately half of the pachytene nuclei lacked both γH2AX domains and RAD51 or DMC1 foci, whereas no nuclei were found without RPA foci (Figure 6A, B). We did not observe any pseudo XY body in nuclei without RAD51 foci, but 13% of the nuclei contained a γH2AX domain but no DMC1 foci (Figure 6A). RPA is known to mark DSB repair spots after RAD51-mediated strand invasion and during homologous recombination, to protect the ssDNA regions generated during this process [55]. This explains the fact that RPA foci are always present in E17.5 oocyte nuclei which are at a mid-meiotic stage and have not yet completed the homologous recombination process at all DSB repair sites. Also, since RPA is engaged in completing recombination at synapsed autosomal sites, a relatively small fraction of the RPA foci colocalizes with pseudo XY bodies. In contrast, most DMC1 and RAD51 foci localize to γH2AX domains, similar to what was found for *Spo11^YF/YF^* oocyte nuclei (Figure 6B), although DMC1 foci are found more frequently and in higher numbers in pseudo XY bodies in *Spo11^+/+^* compared to *Spo11^YF/YF^* oocytes. DMC1 foci colocalized with RAD51 foci when both were present in the pseudo XY body (Figure S6C).

### Pseudo XY bodies overlapping synapsed axes contain RAD51 foci but lack DMC1

**Figure 7:**
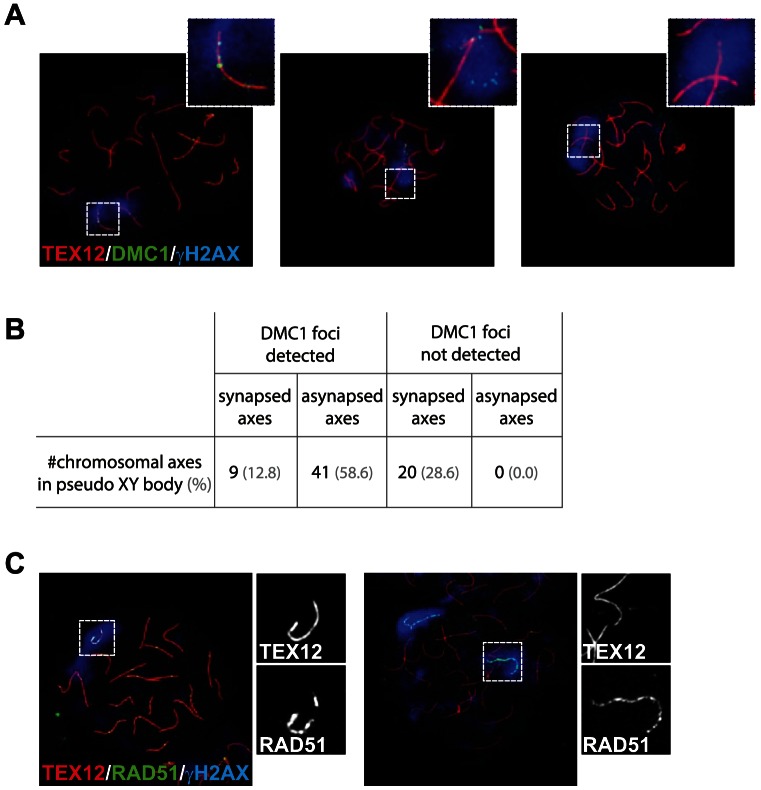
DMC1 preferentially localizes to unsynapsed axes in wild type pachytene oocytes. (A) Triple immunostaining with anti-TEX12 (red), anti-DMC1 (green), and anti-γH2AX (blue) of pachytene oocyte nuclei from E17.5 wild type embryos. DMC1 foci are detected in the pseudo XY body and localize to synapsed axes (left, close-up), or to unsynapsed axes (middle, close-up). The pseudo XY body is often devoid of DMC1 foci (right, close-up). (B) Quantification of the number of synapsed and unsynapsed axes, present in pseudo XY bodies, that are positive or negative for DMC1 foci (n = 70). Percentages are shown in brackets. (C) Triple immunostaining with anti-TEX12 (red), anti-RAD51 (green), and anti-γH2AX (blue) of oocytes from E17.5 wild type embryos. Axis-wide accumulation of RAD51 in the pseudo XY body was observed on both synapsed (left) and unsynapsed (right) axes. Close-ups separately show TEX12 and RAD51 patterns in the pseudo XY body.

**Figure 8:**
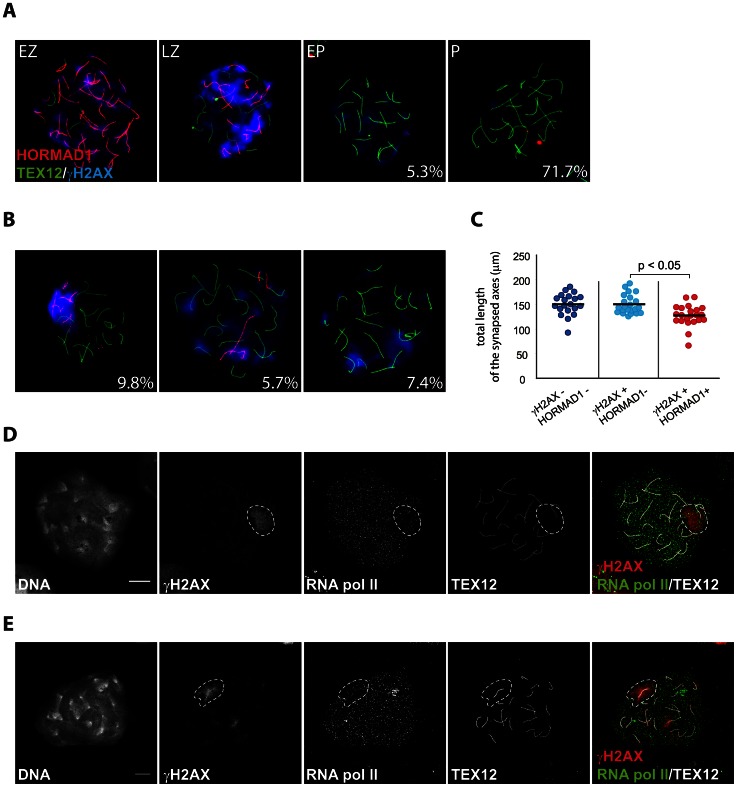
Pseudo XY bodies containing synapsed axes in wild type embryonic oocytes. (A–B) Triple immunostaining with anti-HORMAD1 (red), anti-TEX12 (green) and anti-γH2AX (blue) of oocyte nuclei from E17.5 wild type embryos. In the lower right corner percentages are reported, representing the frequency of each type of cell in the pachytene oocyte population (n = 244). (A) Representative pictures of early zygotene (EZ), late zygotene (LZ), early pachytene (EP) and pachytene (P) oocytes, from left to right. HORMAD1 levels are decreasing while TEX12 accumulates as synapsis progresses. Parallel to the increase of synapsis and HORMAD1 removal, γH2AX accumulation decreases. (B) Representative pictures of pachytene oocytes with a pseudo XY body. HORMAD1 positive axes totally (left picture) or partially (middle picture) colocalize with the pseudo XY body, or are not present (right picture) in the pseudo XY body. (C) Scatter plot of the total length of synapsed axes in E17.5 wild type pachytene oocytes, belonging to the following categories: HORMAD1 and pseudo XY body absent (blue); HORMAD1 absent and pseudo XY body present (light blue); presence of both HORMAD1 and a pseudo XY body. Every dot represents a nucleus. Black bars indicate the mean values. P values for the indicated comparison (Mann-Whitney, two-tailed) are shown in the plot. (D–E) Triple immunostaining with anti-TEX12 (white), anti-RNA polymerase II (green), and anti-γH2AX (red) of pachytene oocytes from E17.5 wild type embryos, imaged with the Zeiss LSM700 confocal microscope. Depletion of RNA pol II can be observed in the area of the the pseudo XY body marked by γH2AX, both when synapsis is complete (D) and when unsynapsed axes (E) are present in this region (E). Size bars represent 10 µm.

Since we observed some differences between the patterns of RAD51 and DMC1 accumulation in pseudo XY bodies of wild type oocytes, we wondered whether pseudo XY bodies that contain both DMC1 and RAD51 foci differ from those that show only RAD51 foci. First, we analysed the relation between DMC1 accumulation, formation of the pseudo XY body and synapsis, using an antibody directed against the central element protein TEX12. The results in Figure 7A and B show that DMC1 foci in oocyte pseudo XY bodies localize mainly (58.6%) on unsynapsed axes (inferred from the absence of TEX12, and placement of DMC1 foci in an axis-like pattern), and rarely (12.8%) on synapsed areas (Figure 7B). It is important to note that 28.6% of oocytes with a pseudo XY body did not show any DMC1 foci (Figure 7A, B) and that all these nuclei were also characterized by complete synapsis (based on the presence of 20 TEX12-positive bivalents) (Figure 7B). In contrast, RAD51 always coats the chromosomal axes of the pseudo XY body, irrespective of synapsis (Figure 7C). These observations prompted us to further analyse the occurrence of pseudo XY bodies in association with complete synapsis. For this, we used an antibody directed against the HORMAD1 protein, together with anti-TEX12 as well as anti-γH2AX to identify the pseudo XY body. As reported previously, HORMAD1 covered all unsynapsed axes at zygotene, and was lost once the cells reached complete synapsis at pachytene [8] (Figure 8A). Conversely, TEX12 gradually accumulated as synapsis progressed, consistent with earlier reports [11] (Figure 8A). When we analysed the pachytene population in more detail, we observed unsynapsed axes that were positive for HORMAD1 in a pseudo XY body in 9.8% of the pachytene nuclei, and another 13.1% that showed partial (5.7%) or no (7.4%) colocalisation of the pseudoXY body with HORMAD1 (Figure 8B). Whenever HORMAD1 was absent from the pseudo XY body, TEX12 was present, indicating complete synapsis. To verify that synapsis was complete in the nuclei that lacked HORMAD1 but contained a pseudo XY body, we measured the total length of synapsed axes, visualized as TEX12 stretches, in pachytene oocyte nuclei. We found that the total SC length was comparable in pachytene oocytes without any HORMAD1 staining, independent of the presence of a pseudo XY body. On the contrary, the total synapsis length was significantly lower in pachytene oocyte nuclei which showed both a pseudo XY body and HORMAD1 (Figure 8C). Finally, to confirm that these pseudo XY bodies elicit true meiotic silencing, despite the absence of asynapsis, we performed a triple staining for RNA polII, TEX12 and γH2AX. As shown in Figure 8D and 8E, RNA polII is depleted from the pseudo XY body, irrespective of synapsis.

## Discussion

### SPO11-dependent DSB formation

A point mutation in the *Spo11* gene that results in the replacement of Tyr 138 by Phe in the catalytic site of the enzyme leads to the absence of detectable SPO11-dependent meiotic DSBs in oocytes and spermatocytes. This observation is in accordance with recent findings of Boateng et al. [43], who analysed a mouse mutant carrying a mutation in the *Spo11* gene that leads to replacement of both Tyr 137 and Tyr138 by Phe.

Although having half the amount of functional SPO11 is sufficient to generate a normal number of crossovers, as evidenced by the analysis of MLH1 foci in *Spo11^+/YF^* spermatocytes and oocytes, the dynamics of DSB induction was clearly altered. The lower number of RAD51 foci that was observed in leptotene *Spo11^+/YF^* oocytes and spermatocytes may indicate that fewer breaks are made. However, near normal numbers of RAD51 foci are observed in zygotene *Spo11^+/YF^* spermatocytes and oocytes. These data are consistent with the homeostatic control mechanism that has been observed in yeast [56] and mouse spermatocytes, allowing maintenance of normal crossover frequencies when the number of DSBs is reduced [57]. In addition, or alternatively, the recently identified feedback mechanism, requiring ATM activity, which regulates the number of breaks that can be formed by SPO11 [58] may ensure that a similar level of DSB formation is reached in the heterozygote, albeit with different kinetics when compared to the wild type.

### SPO11-independent DNA repair foci

In the absence of SPO11, no meiotic DSBs are formed, and accumulation of RAD51, DMC1 and RPA proteins is therefore not expected. Nevertheless we observed significant numbers of RAD51, DMC1 and RPA foci in *Spo11^YF/YF^* and *Spo11^−/−^* oocytes and spermatocytes that preferentially localized in the pseudo XY body, identified on the basis of the γH2AX staining pattern. In *Spo11^YF/YF^* oocytes, we observed a clear increase in the number of RAD51 foci in oocytes at E17.5, compared to oocytes at E16.5. However, the number of DMC1 and RPA foci was much lower than the number of RAD51 foci in these nuclei. The number of DMC1 foci in particular would be expected to follow the same pattern as RAD51, because DMC1 has been reported to participate in the formation of recombination filaments [26]. Nevertheless, it has been recently shown that the dynamics of accumulation of DMC1 and RAD51 are different when extra DSBs are induced by a supplemental copy of the SPO11β-isoform [57]. Cole et al. [57] suggested that, in this situation, the extra DSBs may be more likely to engage in a mitotic pathway of HR repair, and thus less likely to recruit DMC1. In oocytes that completely lack a synaptonemal complex, DMC1 was found to be lost from persistent DSBs, whereas RAD51 foci were still observed [59]. Based on this, it was suggested that DMC1 can only stably associate with meiotic DSBs in the context of synapsed chromatin and normal progression of repair [59]. Our own observations also indicate that DMC1 is lost from SPO11-induced DSB repair sites before RAD51 (data not shown). Together, these observations are in accordance with the notion that the sites that recruit RAD51 foci in E17.5 oocytes can no longer recruit DMC1 with equal efficiency. This may be due to differences in the composition of the repair complexes at (persistent) DSBs in late compared to early pachytene oocytes, or is possibly caused by a drop in the level of DMC1 protein expression.

### Nature of the SPO11-independent DNA repair foci

It is important to establish if the DNA repair foci represent actual sites of DNA damage. The increase in the number of RAD51 foci in oocytes between E16.5 and E17.5 may be due to a DNA-damage independent association of RAD51 to chromosomal axes, or foci formation might be induced by the specific chromatin structure that is formed upon γH2AX formation, which would explain why the foci tend to colocalize in a single subnuclear region. However, we have observed that radiation-induced DSBs, that localize throughout the nucleus, first lead to a nucleus wide accumulation of γH2AX, and subsequently to a more concentrated presence of RAD51 foci and γH2AX in a specific subdomain of the nucleus (the pseudo XY body). In addition, it is known that in spermatocytes that carry autosomes with a pairing problem, meiotic DSBs persist on the unsynapsed regions, in association with MSUC, and these regions then also tend to colocalize with the XY body, indicating that persistent DSBs in the context of MSUC have a tendency to reside together in a single nuclear area [38]. The preferred presence of DMC1 and RPA in addition to RAD51 in the pseudo XY bodies supports the hypothesis of the presence of a DNA damage event. One particular feature of the SPO11-independent repair foci in *Spo11^YF/YF^* oocytes is their inefficient processing. In fact, in oocytes from E17.5 *Spo11^YF/YF^* mice, RAD51 appears to coat unsynapsed axial elements, so that individual foci are no longer clearly observed, indicating that the RAD51 filament formation is not regulated as in a normal homologous DSB repair event. Upon replacement of RPA by RAD51/DMC1, and subsequent persistence of a DSB without further processing to a recombination intermediate, such an axis-wide pattern for RAD51 may develop, possibly due to an abnormal regulation of the foci dynamics, compared to conventional DSB repair events. The spreading of RAD51 along axial elements may result from spreading of RAD51 onto double-stranded DNA, a phenomenon that has also been described for persistent DSBs in yeast [60]. Based upon these considerations, we favour the conclusion that the SPO11-independent DNA repair foci represent true sites of persistent DNA damage.

### Origin of SPO11-independent DNA repair foci

To explain what might cause spontaneous DNA damage in *Spo11^YF/YF^* and knockout spermatocytes and oocytes, and possibly also in wild type meiocytes, different mechanisms can be proposed. First, during S phase in somatic cells, and most likely also in meiocytes, DSBs can form at stalled replication forks. In human cells, 50 endogenous DSBs have been proposed to occur in every cell cycle [61]. Most of these DSBs will be repaired before the cells enter G2, but some may persist, and the number of persisting breaks appears to vary between different cell types [62], [63]. A second mechanism that could generate endogenous DSBs is transcription-associated recombination (TAR). The causes of DSBs that form in association with ongoing gene transcription are thought to be related either to generation of stalled replication forks in association with transcription, or to increased accessibility of DNA during transcription, making it more vulnerable to DNA-damaging agents (reviewed by [64], [65]). Meiocytes are post S phase cells, and leptotene, zygotene, and early pachytene spermatocytes and oocytes display a low level of RNA synthesis, making TAR an unlikely source of RAD51 foci in these cells [66], [67]. A third possible endogenous source of DSBs is impaired topoisomerase II activity. Inhibition of topoisomerase II activity in pachytene spermatocytes has been found to result in DSB formation, indicating that topoisomerase II is indeed functional in meiocytes [68]. Fourth, endonuclease activity of ORF2, encoded by *Line1* transposons, generates DSBs during the transposition of mobile elements in the genome [69]–[71]. Derepression of transposons has been shown to cause SPO11-independent DNA damage in *Mael* mutant spermatocytes [72]. In wild type oocytes and spermatocytes, transcription of *Line1* elements is transiently derepressed at the onset of meiosis [73]. Finally, we cannot exclude that DNA damage may occur as a result of unknown environmental or endogenous factors such as reactive oxygen species (ROS). ROS generation has been described for normal rat spermatocytes [74], but it is not clear to what extent such damage also results in RAD51 foci formation.

In *Spo11^YF/YF^* spermatocytes, it appears most likely that some or all of the SPO11-independent RAD51 foci result from carry-over of spontaneous DSBs that were induced in the previous S phase. In oocytes this may also occur, and the observed *de novo* generation of RAD51 foci post S phase in *Spo11^YF/YF^* oocytes indicates that (additional) spontaneous DSBs in oocytes may arise either from impaired topoisomerase II activity or from ORF2 mediated endonuclease activity in cells that should have progressed already to pachytene. Such SPO11-independent DNA damage may also be induced in wild type pachytene oocytes, but the close proximity of the homologous template in these oocytes may facilitate homologous recombination repair of most of the *de novo* induced DNA damage. In *Spo11^YF/YF^* oocytes the appropriate template for repair is not directly available due to almost complete lack of homologous chromosome pairing. This difference in homologous template availability readily explains the higher relative frequency of pseudo XY body formation in *Spo11^YF/YF^* oocytes compared to oocytes from wild type or heterozygote littermate controls. At present, it is not clear whether the persistent repair foci are resolved at some later time point, or whether the persistent presence of these foci and the associated γH2AX signaling triggers a checkpoint that induces apoptosis. Daniel et al. [22] reported increased apoptosis of oocytes in ovaries of newborn *Spo11* knockout mice compared to controls. In addition, it has been reported that only 10–20% of the normal number of oocytes is present in *Spo11* knockout ovaries at postnatal days 4 and day 8 [34], [75]. This percentage nicely corresponds to the 19% of oocytes that do not contain a pseudo XY body at E17.5 in our *Spo11^YF/YF^* model. However, although these data confirm that oocytes with a pseudo XY body are lost shortly after birth, cell death may also be caused by a so-called synapsis checkpoint, mediated by HORMAD proteins, rather than by a DNA repair checkpoint [22], [23], [76].

### Two types of pseudo XY bodies in wild type oocytes

Our analyses of RAD51 and DMC1 foci in relation to MSUC and synapsis in pachytene oocytes from *Spo11^+/YF^* and wild type E17.5 embryos has shown that two different types of equally silenced pseudo XY bodies exist in wild type pachytene oocytes. Approximately two-third of the pseudo XY bodies accumulate DMC1 as well as RAD51 and form on unsynapsed chromatin (Type I), whereas one-third accumulate RAD51, but little or no DMC1, and form on synapsed chromatin (Type II). We propose that the Type I pseudo XY bodies represent sites that contain persistent SPO11-induced DSBs in areas that failed to synapse, whereas the Type II pseudo XY bodies represent sites where SPO11-independent damage has persisted that elicited a MSUC response, independent of synapsis.

### Persistent DSBs nucleate meiotic silencing

**Figure 9:**
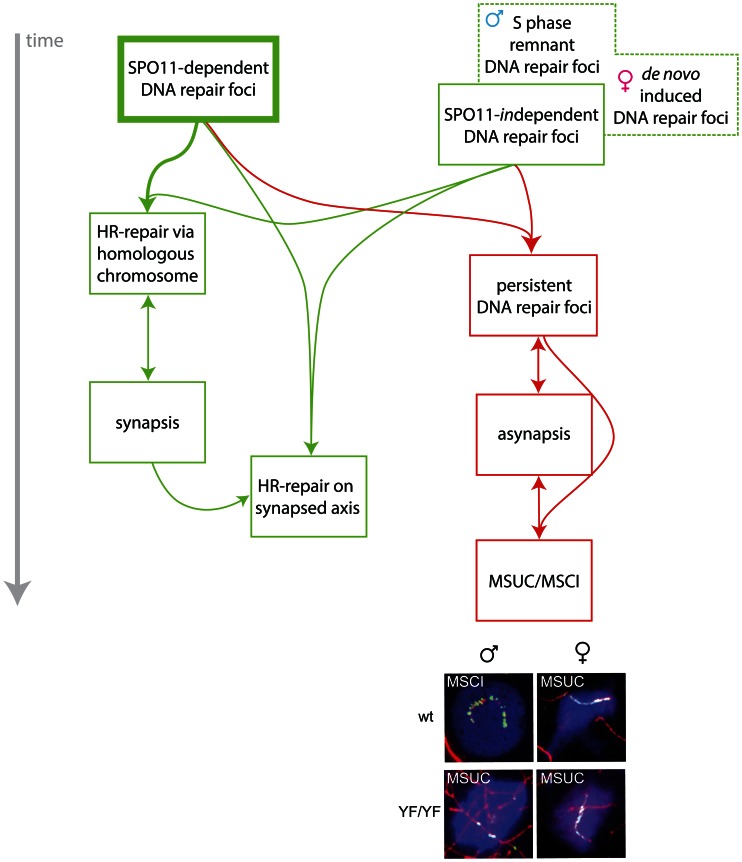
Model for the roles of SPO11-dependent and -independent meiotic DSBs in synapsis and meiotic silencing. In spermatocytes and oocytes, SPO11 generates many meiotic DSBs which are repaired via homologous recombination (HR). This repair process requires the use of the homologous chromosome as a repair template and promotes homologous chromosome synapsis. Once the homologs are in close juxtaposition, synapsis proceeds. Subsequently, repair may occur faster, perhaps now allowing the use of both the homologous chromosome and the sister chromatid as a template for repair. In the absence of a repair template, DSBs persist, inhibiting synapsis between non-homologous partners, although some repair via the sister chromatid on chromosomes that are not synapsed is not excluded. Conversely, asynapsis also contributes to the persistence of DSBs when repair via the sister chromatid remains suppressed. The presence of persistent DSBs on unsynapsed axes, may lead to local accumulation of γH2AX and activate a positive feedback mechanism that involves HORMAD activation, followed by recruitment of ATR, which will lead to rapid spreading of a signal along the unsynapsed axes that will then induce accumulation of γH2AX on the chromatin surrounding these axes. This process always occurs on the XY pair in spermatocytes and leads to MSCI. In the absence of SPO11-induced DSBs, SPO11-independent DNA damage nucleates MSUC via the same mechanism. In spermatocytes, SPO11-independent DNA repair foci may represent remnant DSBs that have formed during the premeiotic S phase. In oocytes (both wild type and SPO11-mutant), SPO11-independent DNA repair foci form late, at a time point corresponding to early pachytene. Such *de novo* induced DNA repair foci, most likely caused by some form of DNA damage, together with unrepaired SPO11-induced DSBs, and frequently in combination with occasional asynapsis, result in γH2AX accumulation and activation of MSUC. Representative images of the (pseudo) XY body in male and female nuclei from wild type (wt) and *Spo11^YF/YF^* nuclei are shown. The immunostainings show SYCP3 (red), γH2AX (blue) and RAD51 (green).

The percentage of cells with γH2AX accumulation in a pseudo XY body is highly reduced in *Spo11^−/−^ Hormad1^−/−^* or *Spo11^−/−^ Hormad2^−/−^* double mutant spermatocytes [22], [23]. This illustrates the important role of HORMAD proteins in the MSUC response. Yet the localization of HORMAD1 to all unsynapsed chromatin in *Spo11* knockout spermatocytes [22], [23]), and the presence of some nuclei with a proper MSUC response in *Spo11^−/−^ Hormad1^−/−^* spermatocytes indicate that, apart from HORMAD proteins, an additional localizing event is needed for pseudo XY body nucleation. Taken together, these and our observations support the hypothesis that both asynapsis, detected by HORMADs, and persistent SPO11-independent DNA repair foci are involved in the induction of H2AX phosphorylation and the establishment of meiotic silencing in pseudo XY bodies in *Spo11^YF/YF^* oocyte nuclei. We would like to propose that MSCI in wild type spermatocytes is then also triggered by both persistent DSBs, in this case SPO11-dependent, and the presence of unsynapsed chromatin (schematically presented in Figure 9).

If RAD51 accumulation is as extensive as observed in pseudo XY bodies in oocytes, HORMADs may not even be required, and enough ATR may be recruited by the DNA repair machinery itself, to elicit the MSUC response, as indicated by the existence of pseudo XY bodies that lack HORMAD1 in oocytes.

Despite the more prominent RAD51 accumulation on axes of the pseudo XY body in oocytes as compared to spermatocytes, we propose that the mechanism of pseudo XY body formation in *Spo11^YF/YF^* spermatocytes occurs in a similar fashion. The differences in the pattern of RAD51 accumulation may be caused by the fact that *Spo11^YF/YF^* spermatocytes are eliminated at stage IV of the spermatogenic cycle, whereas *Spo11^YF/YF^* oocytes appear to proceed normally throughout the stage that should correspond to pachytene and are eliminated later [34]. Perhaps, the few spontaneous DSBs in *Spo11^YF/YF^* spermatocytes modulate the MSUC response in a slightly different way, compared to the responses elicited by the more extensive accumulation of endogenous DSBs in *Spo11^YF/YF^* oocytes. Still, the MSUC response in both *Spo11^YF/YF^* spermatocytes and oocytes is characterized by the same intense γH2AX accumulation and by the presence of RAD51/DMC1 and RPA foci. It is interesting to note that such foci can also be observed on the unsynapsed axes of the X chromosome in wild type spermatocytes, as a hallmark of persistent DSBs. HORMAD proteins may be instrumental to spread the MSUC response along the chromosomal axes into areas that lack persistent DSBs, such as the Y chromosome. In somatic cells, formation of γH2AX chromatin domains has also been coupled to transcriptional silencing, in the context of radiation-induced damage [16]. More recently, Shanbhag et al. [39] analysed the effect of persistence of an endonuclease-dependent DSB on transcriptional activity in the neighbouring genes. They observed that H2AX phosphorylation spreads along the DNA surrounding the DSB, and that the accumulation of this histone modification correlated with reduction of RNA polymerase II activity. Persistent DSBs were shown to trigger the silencing of neighbouring genes, and the mechanism was termed DSB-induced silencing *in cis* (DISC) [39]. This mechanism, that occurs in somatic cells, might have some aspects in common with MSUC and MSCI in meiocytes.

In conclusion, this study has revealed the presence of SPO11-independent DNA repair foci in oocytes and spermatocytes. In addition, we show that unrepaired DSBs most likely are the initial trigger of both MSCI and MSUC in spermatocytes and oocytes. For wild type oocytes, the possible presence of *de novo* induced DNA damage in a substantial part of the oocyte population may contribute to the massive loss of such oocytes around birth. For spermatocytes, the few SPO11-independent breaks that are present will most likely be rapidly repaired once homologous chromosome pairing is obtained with the help of the 200 or more SPO11-induced DSBs. The MSUC and MSCI response may be less unique than previously thought, and actually represent an extreme and adapted form of DISC. Therefore, knowledge about the molecular basis of meiotic silencing may also be relevant for our understanding of DNA damage-induced chromatin modifications in somatic cells.

## Materials and Methods

### Ethics statement

All animal experiments were approved by the local animal experiments committee DEC Consult.

All animals were housed in IVC cages under supervision of the Animal Welfare Officer. Any discomfort of animals was daily scored by the animal caretakers. No more than mild or moderate discomfort of animals was expected from the treatments, and no unexpected discomfort was observed.

### Mice

All animal experiments were approved by the animal experiments committee DEC-Consult.

*Spo11* mutant mice were generated through a two-step recombination strategy as described by Soukharev et al., [77]. First, two heterospecific *lox* sites flanking the selectable marker hygromycin, replacing exons 4–8, were placed in the *Spo11* gene, in ES cells by homologous recombination. Next, a targeting vector containing the same heterospecific *lox* sites flanking exon 4–8 of *Spo11* with the point mutation generating Y138F at exon 4 was used to replace the selection marker by a site-specific double cross-over event (Figure S1A). The final modified *Spo11* locus carries a *loxP* site between exons 3 and 4, the point mutation generating Y138F at exon 4, and a *lox511* site between exons 8 and 9. ES cells carrying a single modified *Spo11* allele were used for blastocyst injection to generate chimeras, and heterozygotes were obtained upon germ line transmission of the mutated allele. Correct targeting was verified using Southern blotting with 5′and 3′probes outside the targeted region (Figure S1B), and sequencing (Figure S1C). This *Spo11* allele has been registered at Mouse Genome Informatics (MGI) as *Spo11<tm1Bdm>* (Allele Accession ID: MGI:5432496).

Wild type, heterozygote and homozygote *Spo11* mutant mice were kept on a FVB background. To genotype the animals, the following primers were used: forward, 5′CTGGTCGATGCAGATCCCTACGG3′; reversed, 5′TAGATGCACATTATCTCGATGCC3′ (Figure S1B)

*Spo11* knockout mice carried the *Spo11^tm1M^* allele described in [34].

For the analysis of radiation-induced DSBs in spermatocytes, *Spo11^YF/YF^* male adult mice were exposed to 5Gy whole body radiation and sacrificed 1 h, 48 h, and 120 h after the treatment to collect the testes.

### Antibodies

For primary antibodies, we used mouse monoclonal antibodies anti-phosphorylated H2AX, anti-BRCA1, anti-TOPBP1, anti-MDC1, anti-phospho H2AX (all from Upstate), anti-DMC1 (DMC1-specific), anti-RAD51, anti-RNA Polymerase II (all from Abcam); rabbit polyclonal antibodies anti-RAD51 (recognizing both DMC1 and RAD51) [78], anti-RPA (gift from P. De Boer, described in Schaarmidt et al., ([79]), anti-SYCP3 (gift from C. Heyting), anti-HORMAD1 (gift from A. Tóth) and anti-phosphorylated H2AX (Upstate); rat polyclonal anti-SYCP3 [80]; guinea pig anti-TEX12 (gift from Christer Höög). SPO11 antibody (Spo11L56S9) was raised from rabbits immunized with GST-Spo11α produced by the service of recombinant protein of CRBM (UMR5237-CNRS). For secondary antibodies, we used a goat anti-rabbit IgG alexa 405/488/546/633, goat anti-mouse alexa IgG 350/488/546/633, goat anti-rat IgG alexa 546, goat anti-guinea pig 405/555 (Molecular Probes).

### Expression analysis

RNA was extracted and reverse transcribed according to standard procedures. PCR amplifications were performed with forward primer 5′AATAGTCGAGAAGGATGCAACA3′and reversed primer 5′TAGATGCACATTATCTCGATGC3′

Immunoprecipitations were carried out with rabbit polyclonal anti-SPO11 antibody, followed by western blot detection with the same primary antibody and Trueblot secondary antibody (eBioscience).

### Histology

Testes were fixed and stained with hematoxilin and eosin using standard histological methods.

### Meiotic spread nuclei preparations and immunocytochemistry

Testis tissues were processed to obtain spread nuclei for immunocytochemistry as described by Peters et al. (1997) [81]. Spread nuclei of spermatocytes were stained with antibodies mentioned above. Before incubation with antibodies, slides were washed in PBS (3×10 min), and non-specific sites were blocked with 0.5% w/v BSA and 0.5% w/v milk powder in PBS. Primary antibodies were diluted in 10% w/v BSA in PBS, and incubations were overnight at room temperature in a humid chamber. Subsequently, slides were washed (3×10 min) in PBS, blocked in 10% v/v normal goat serum (Sigma) in blocking buffer (supernatant of 5% w/v milk powder in PBS centrifuged at 14,000 rpm for 10 min), and incubated with secondary antibodies in 10% normal goat serum in blocking buffer at room temperature for 2 hours. Finally, slides were washed (3×10 min) in PBS (in the dark) and embedded in Prolong Gold with or without DAPI (invitrogen). Fluorescent images were observed by using a fluorescence microscope (Axioplan 2; Carl Zeiss) equipped with a digital camera (Coolsnap-Pro; Photometrics). To distinguish zygotenes from aberrant pachytenes, we used specific parameters defined in Figure S9. Aberrant pachytene oocytes,have also been described in previous publications [7], [51], and are characterized by the presence of one to three chromosome pairs lacking synapsis. We also included rare nuclei in which some chromosomes are entangled and not fully synapsed. Normal (late) zygotene nuclei are characterized by a higher proportion of homologs that have not completed synapsis, compared to what is observed in the aberrant pachytenes, and SYCP1/TEX12 patches can be observed which have not yet converged to become a single complete central element. In addition to specific characteristics of the SC, the labelling patterns of the repair associated recombinase RAD51 and phosphorylated H2AX are also helpful to distinguish late zygotenes from aberrant pachytenes. Single, isolated RAD51 foci are observed in zygotene nuclei, whereas multiple closely adjacent foci are present in aberrant pachytenes. H2AX phosphorylation,occurs in a nucleus-wide pattern at zygotene. In contrast, aberrant pachytene oocytes have one to three bright and defined γH2AX domains.

Fluorescent images were taken under identical conditions for all slides, and images were analyzed using the ImageJ (Fiji) software (Rasband, W.S., ImageJ, U.S. National Institutes of Health, Bethesda, Maryland, USA [http://rsb.info.nih.gov/ij/]). Confocal imaging was performed on a Zeiss LSM700 microscope (Carl Zeiss, Jena): we used 63× oil immersion objective lens (N.A. 1.4), pinhole 1AU. DAPI was excited at 405 nm and imaged with a short pass filter (SP) 490 nm; Alexa 488 was excited at 490 nm and imaged SP 555 nm; Alexa 546 was excited at 555 nm and imaged SP 640 nm; Alexa 633 was excited at 639 nm and for the imaging no filter was required.

### Quantification of repair foci, synaptonemal complex length, and RNA pol II intensity

Imaging of nuclei immunostained for RAD51 or DMC1 or RPA and SYCP3 was performed with the same exposure time for each nucleus. Images were analysed without any manipulation of brightness and contrast. Foci were subsequently counted using Image J software, including the Fiji plug-in. We used the analyze particles function and set the threshold manually, in order to include the smallest visible focus in the analysis. The average area of one RAD51 focus was assessed to be 40–50 pixels, therefore foci with an area larger than 100 pixels were counted as multiple foci to allow approximate quantification of RAD51 foci also when it was observed as a continuous signal along the axial elements.

Measurement of synaptonemal complex length was performed using a homemade ImageJ macro. The macro generates a skeletonized image of the original picture and measures the length of that skeleton.

Relative quantification of the RNA polII levels in the (pseudo) XY body was performed comparing the average intensity per pixel area in the γH2AX domain with the average intensity in a non-heterochromatic nuclear area of the same shape and size.

## Supporting Information

Figure S1 **Generation of** ***Spo11^YF/YF^*** **mice.** (A) Intron/exon structure of the *Spo11* gene. Step I: homologous recombination using a NotI fragment that replaces exons 5–9 and part of the flanking introns for a HYG/TK positive/negative selectable marker cassette and two heterologous lox sites, loxC33 and lox511. Step II: Cre-mediated cassette exchange using a donor plasmid that replaces the HYG/TK cassette for a mutated *Spo11* fragment carrying the F138 codon in exon 5. (B) (left) Southern blot to visualize a diagnostic BclI fragment using the 5′ probe as indicated in A. Correct integration enlarges the BclI fragment from 12 kb to 18 kb (right). PCR using primers in exon 9 and 10 distinguishes the wild-type allele (394 bp) from the mutant allele carrying the lox511 site in intron 9 (482 bp). (C) Sequencing of *Spo11* cDNA from wild-type (+/+), heterozygote (+/YF) and homozygote (YF/YF) knock-in mice. The A-T mutation that changes the TAC codon for Tyrosine into a TTC codon for Phenylalanin is boxed. (D) RT-PCR to analyse mRNA expression using testis RNA from 15 day-old-mice, wild-type (+/+), heterozygote (+/YF) and homozygote (YF/YF). Using a forward primer in exon 8 and a reversed primer in exon 10, two splice variants can be detected in wild type and knock-in testes (drawing on the left). Due to the fact that a Loxp site resides between exon 9 and 10, the splice variant that includes these intronic sequences is larger in the *Spo11^YF/YF^*. (E) Immunoprecipitation and detection of SPO11 in testis extracts from adult wild type (+/+) and *Spo11* knockout (−/−) and 16 days old wild-type (+/+), heterozygote (+/YF) and homozygote (YF/YF) knock-in mice (16d). The positions of the two SPO11 isoforms (β and α) are shown. M: molecular weight marker.
(TIF)

Figure S2 **Spermatogenesis and oogenesis are blocked at a zygotene-like stage in** ***Spo11^YF/YF^*** **mice.** (A) Hematoxylin-eosin staining of testis from adult wild type (+/+) and *Spo11^YF^* ^***/YF***^ (YF/YF) mice. Immunostaining of spread nuclei of spermatocytes (B) and oocytes (C) of wild-type (+/+), *Spo11^+^* ^***/YF***^ (+/YF) and *Spo11^YF^* ^***/YF***^ (YF/YF) mice. For wild type and heterozygote mice, leptotene, zygotene and pachytene nuclei are shown. For the *Spo11^YF/YF^* mice, leptotene, zygotene and late zygotene -like nuclei are shown.
(TIF)

Figure S3 **Pattern of RAD51 foci in E17.5 oocyte nuclei is confirmed by ab1837 Abcam antibody.** (A–B) Double immunostaining of pseudo XY body-positive *Spo11^YF/YF^* (A) and *Spo11^+/+^* (B) E17.5 oocyte nuclei with anti-SYCP3 (red), anti-RAD51 (green), and anti-γH2AX (blue).
(TIF)

Figure S4 **RAD51 foci in** ***Spo11^−/−^*** **spermatocyte and E17.5 oocyte nuclei.** (A–B) Double immunostaining of *Spo11^−/−^* spermatocyte (A) and E17.5 oocyte (B) nuclei with anti-SYCP3 (red) and anti-RAD51 (green). Arrows indicate RAD51 foci (A) and axis-wide RAD51 accumulation (B).
(TIF)

Figure S5 **Pseudo XY body in** ***Spo11^YF/YF^*** **spermatocytes.** (A–D) Double immunostaining of *Spo11^YF/YF^* spermatocytes with anti-SYCP3 (red) and different DNA repair proteins or histone modifications (green). Antibodies used for immunostaining are indicated. Arrows mark the localization of the pseudo XY body.
(TIF)

Figure S6 **RAD51 and DMC1 foci colocalize in mouse meiocytes.** (A–C) Immunostaining of *Spo11^YF/YF^* spermatocyte (A), *Spo11^YF/YF^* E17.5 oocyte (B), and *Spo11^+/+^* E17.5 oocyte (C) nuclei with anti-RAD51 (red), anti-DMC1 (green) and anti-γH2AX (blue). Close-ups show RAD51 and DMC1 foci in the area of the pseudo XY body next to every nucleus: red and green channels overlaid (top) and offset (bottom).
(TIF)

Figure S7 **Limited colocalization of RPA and DMC1 during spermatogenesis.** Mouse spermatocyte nuclei were stained with anti-DMC1 (green) and anti-RPA (red). DAPI was used to visualize the DNA and stage spermatocytes from leptotene (L) through zygotene (Z) to pachytene (P). Early to late pachytene spermatocytes were distinguished based on the conformation of the X and Y chromosomal axes, that were visible in the DAPI image. Consecutive prophase stages are shown from top to bottom. Dashed circles show the nuclear area of the sex body. Both RPA and DMC1 are very abundant at the onset of meiosis. Most likely, RPA is first loaded on the processed 3′ ssDNA strands, and then replaced by DMC1 and RAD51. Starting from late zygotene onwards, DMC1 foci decrease in number, presumably because the recombinase has accomplished its function and its presence is no longer needed. At the same time RPA is recruited again to protect areas of ssDNA generated during the recombination process. Note that at early pachytene, the X chromosome is clearly enriched for DMC1 but not for RPA foci. However, RPA foci increase on the X at late pachytene, when almost all DMC1 and autosomal RPA foci have disappeared. In general, colocalization of DMC1 and RPA is only sporadically observed at all stages examined.
(TIF)

Figure S8 **Correlation between DNA repair markers and pseudo XY body formation in** ***Spo11^−/−^*** **spermatocytes and oocytes.** (A) Immunostaining of spermatocyte (left) and E17.5 oocyte (right) nuclei from *Spo11^−/−^* animals with anti-SYCP3 (red), anti-γH2AX (blue) and anti-RAD51 (green, upper panel) or anti-DMC1 (green, middle panel) or anti-RPA (green, lower panel). SPO11-independent foci are observed in the same pattern as in *Spo11^YF/YF^* meiocytes (B) Quantification of pseudo XY body and DNA repair marker foci positive spermatocytes (n = 120) in *Spo11^−/−^* animals. Nuclei with four different staining patterns were distinguished as indicated by the cartoons above the colums. Numbers indicate percentages. (C) The number of RAD51, DMC1, and RPA foci was counted in the subpopulation of spermatocytes showing both foci and a pseudo XY body. The average total number of foci is reported in the first column of the table. The percentage of colocalization of RAD51, DMC1 or RPA foci with pseudo XY body is shown in the second column. The percentage of pseudo XY bodies which also contain at least one focus of RAD51, DMC1 or RPA is reported in the third column.
(EPS)

Figure S9 **Parameters to discriminate between zygotene and aberrant pachytene wild type oocytes.** (A) Summary of the applied parameters to discriminate between zygotene oocytes and aberrant pachytene oocytes. Patterns of SYCP3, SYCP1 (or TEX12), γH2AX, and RAD51 are described for both categories. (B) Representative images of the described oocyte categories. Oocytes were immunostained for TEX12 (red), RAD51 (green), and γH2AX (blue).
(TIF)
