# Supplementary figures and images for: SPO11-Independent DNA Repair Foci and Their Role in Meiotic Silencing
Source: PLoS Genet. 2013 Jun 6;9(6):e1003538. doi: 10.1371/journal.pgen.1003538 (PMC3675022; doi:10.1371/journal.pgen.1003538)

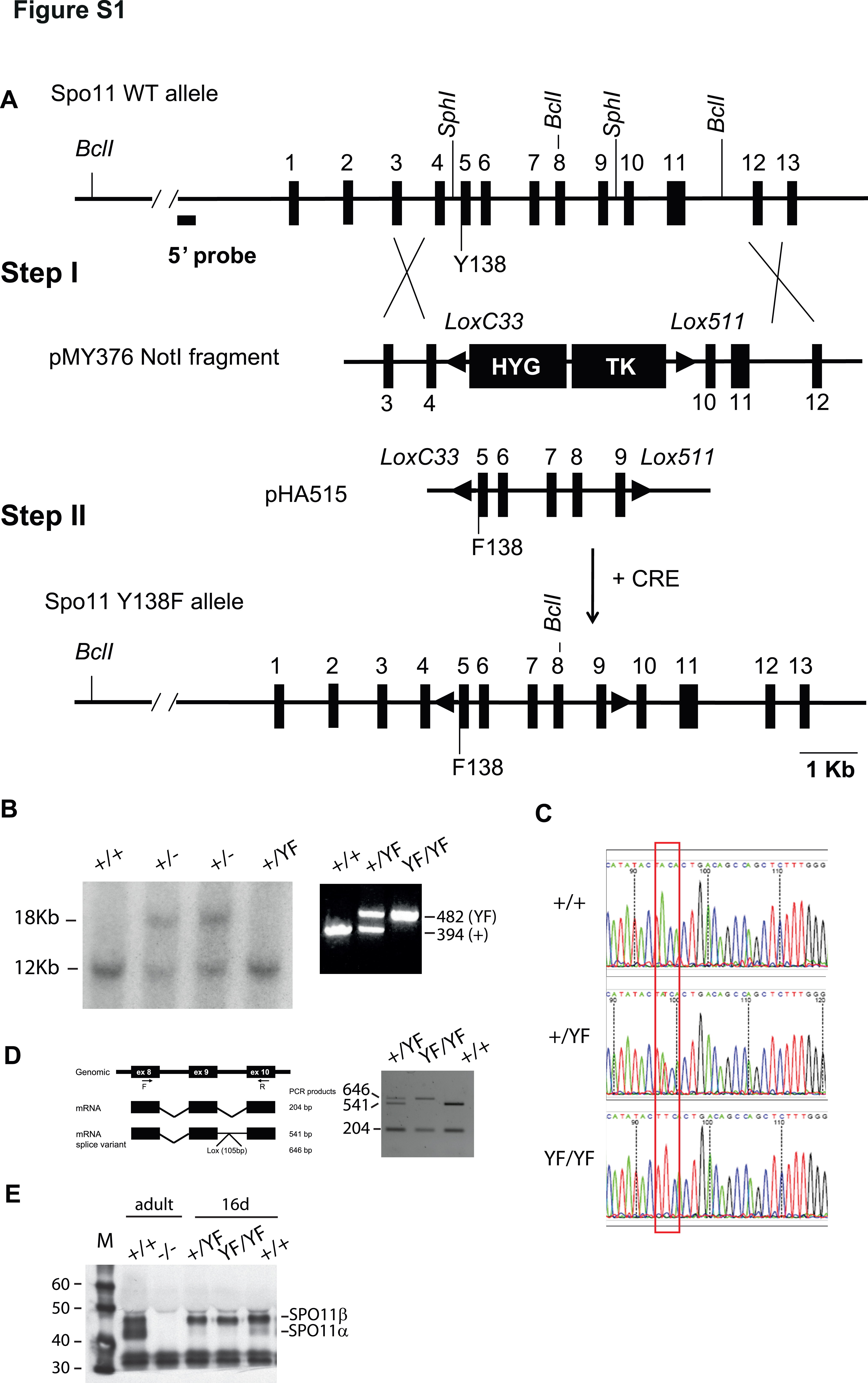

Supplement: Figure S1 — Generation of Spo11YF/YF mice. (A) Intron/exon structure of the Spo11 gene. Step I: homologous recombination using a NotI fragment that replaces exons 5–9 and part of the flanking introns for a HYG/TK positive/negative selectable marker cassette and two heterologous lox sites, loxC33 and lox511. Step II: Cre-mediated cassette exchange using a donor plasmid that replaces the HYG/TK cassette for a mutated Spo11 fragment carrying the F138 codon in exon 5. (B) (left) Southern blot to visualize a diagnostic BclI fragment using the 5′ probe as indicated in A. Correct integration enlarges the BclI fragment from 12 kb to 18 kb (right). PCR using primers in exon 9 and 10 distinguishes the wild-type allele (394 bp) from the mutant allele carrying the lox511 site in intron 9 (482 bp). (C) Sequencing of Spo11 cDNA from wild-type (+/+), heterozygote (+/YF) and homozygote (YF/YF) knock-in mice. The A-T mutation that changes the TAC codon for Tyrosine into a TTC codon for Phenylalanin is boxed. (D) RT-PCR to analyse mRNA expression using testis RNA from 15 day-old-mice, wild-type (+/+), heterozygote (+/YF) and homozygote (YF/YF). Using a forward primer in exon 8 and a reversed primer in exon 10, two splice variants can be detected in wild type and knock-in testes (drawing on the left). Due to the fact that a Loxp site resides between exon 9 and 10, the splice variant that includes these intronic sequences is larger in the Spo11YF/YF. (E) Immunoprecipitation and detection of SPO11 in testis extracts from adult wild type (+/+) and Spo11 knockout (−/−) and 16 days old wild-type (+/+), heterozygote (+/YF) and homozygote (YF/YF) knock-in mice (16d). The positions of the two SPO11 isoforms (β and α) are shown. M: molecular weight marker. (TIF) [file pgen.1003538.s001.tif]

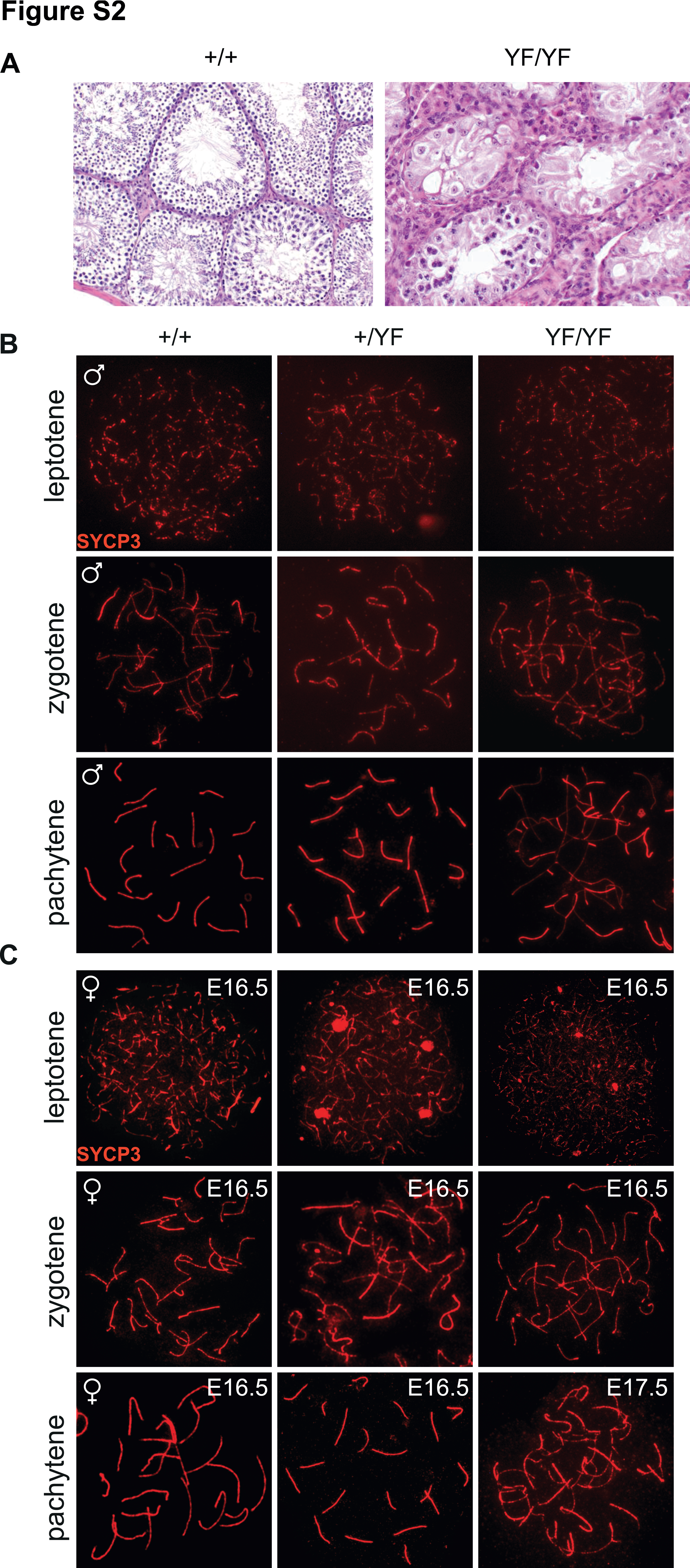

Supplement: Figure S2 — Spermatogenesis and oogenesis are blocked at a zygotene-like stage in Spo11YF/YF mice. (A) Hematoxylin-eosin staining of testis from adult wild type (+/+) and Spo11YF /YF (YF/YF) mice. Immunostaining of spread nuclei of spermatocytes (B) and oocytes (C) of wild-type (+/+), Spo11+ /YF (+/YF) and Spo11YF /YF (YF/YF) mice. For wild type and heterozygote mice, leptotene, zygotene and pachytene nuclei are shown. For the Spo11YF/YF mice, leptotene, zygotene and late zygotene -like nuclei are shown. (TIF) [file pgen.1003538.s002.tif]

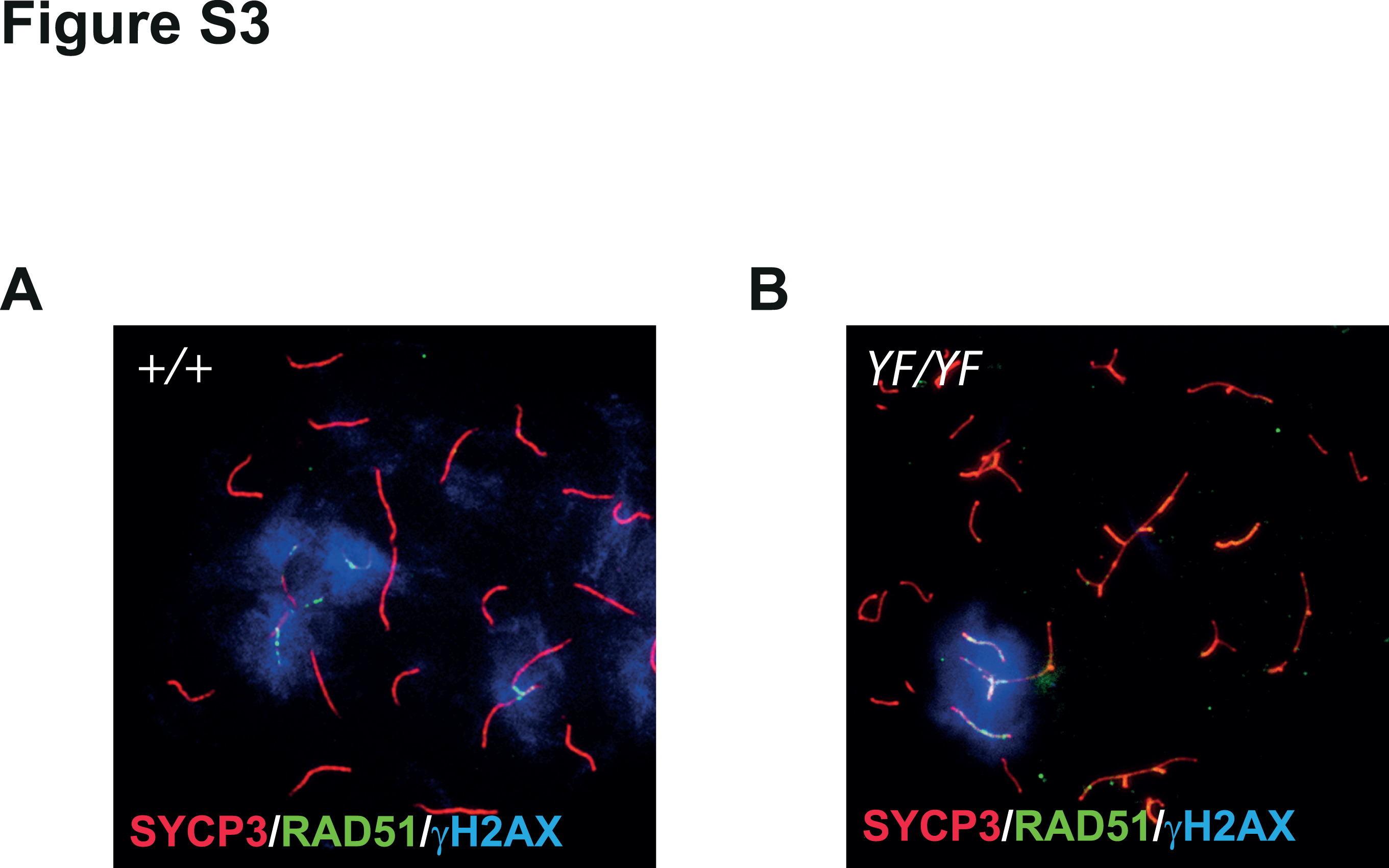

Supplement: Figure S3 — Pattern of RAD51 foci in E17.5 oocyte nuclei is confirmed by ab1837 Abcam antibody. (A–B) Double immunostaining of pseudo XY body-positive Spo11YF/YF (A) and Spo11+/+ (B) E17.5 oocyte nuclei with anti-SYCP3 (red), anti-RAD51 (green), and anti-γH2AX (blue). (TIF) [file pgen.1003538.s003.tif]

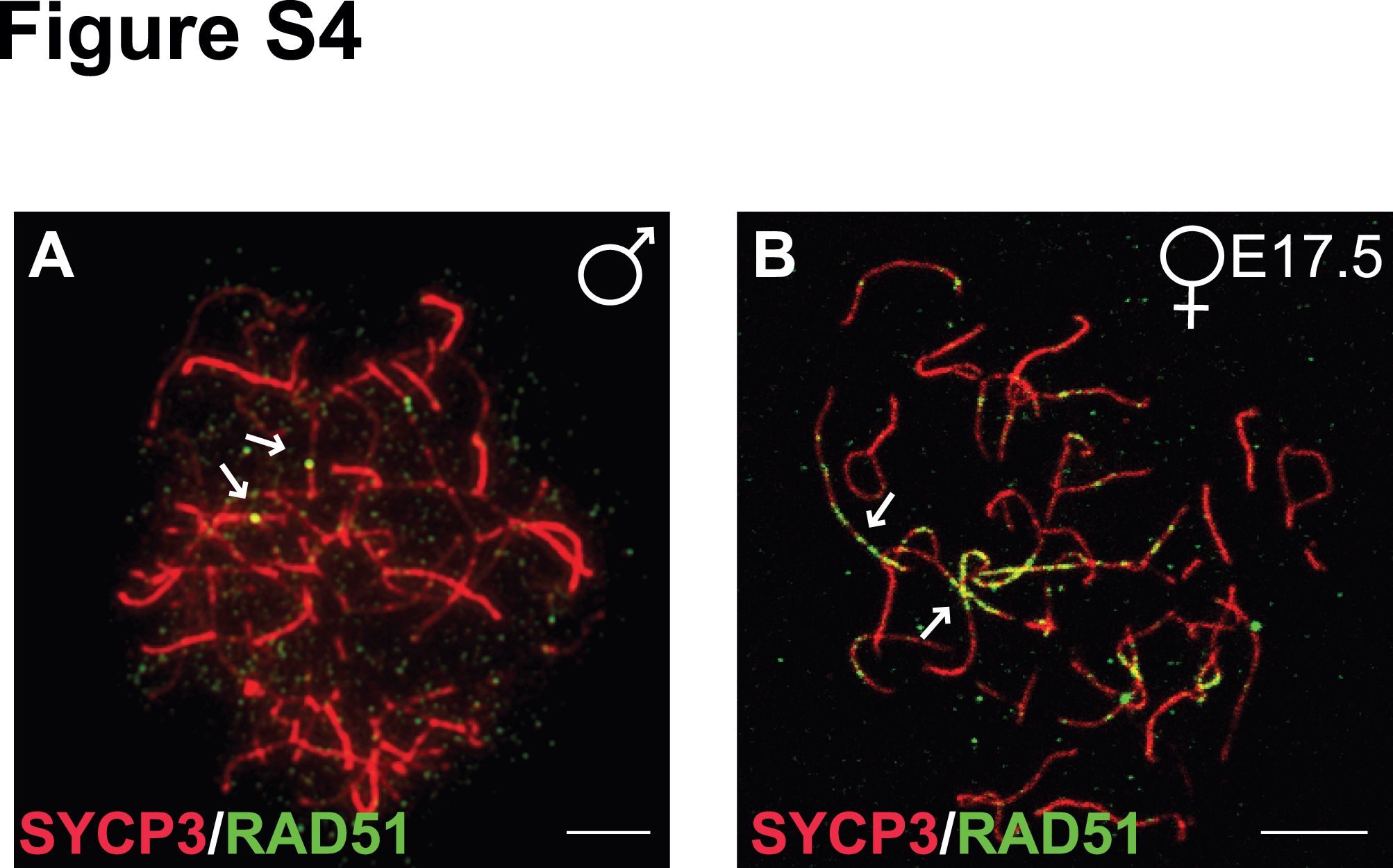

Supplement: Figure S4 — RAD51 foci in Spo11−/− spermatocyte and E17.5 oocyte nuclei. (A–B) Double immunostaining of Spo11−/− spermatocyte (A) and E17.5 oocyte (B) nuclei with anti-SYCP3 (red) and anti-RAD51 (green). Arrows indicate RAD51 foci (A) and axis-wide RAD51 accumulation (B). (TIF) [file pgen.1003538.s004.tif]

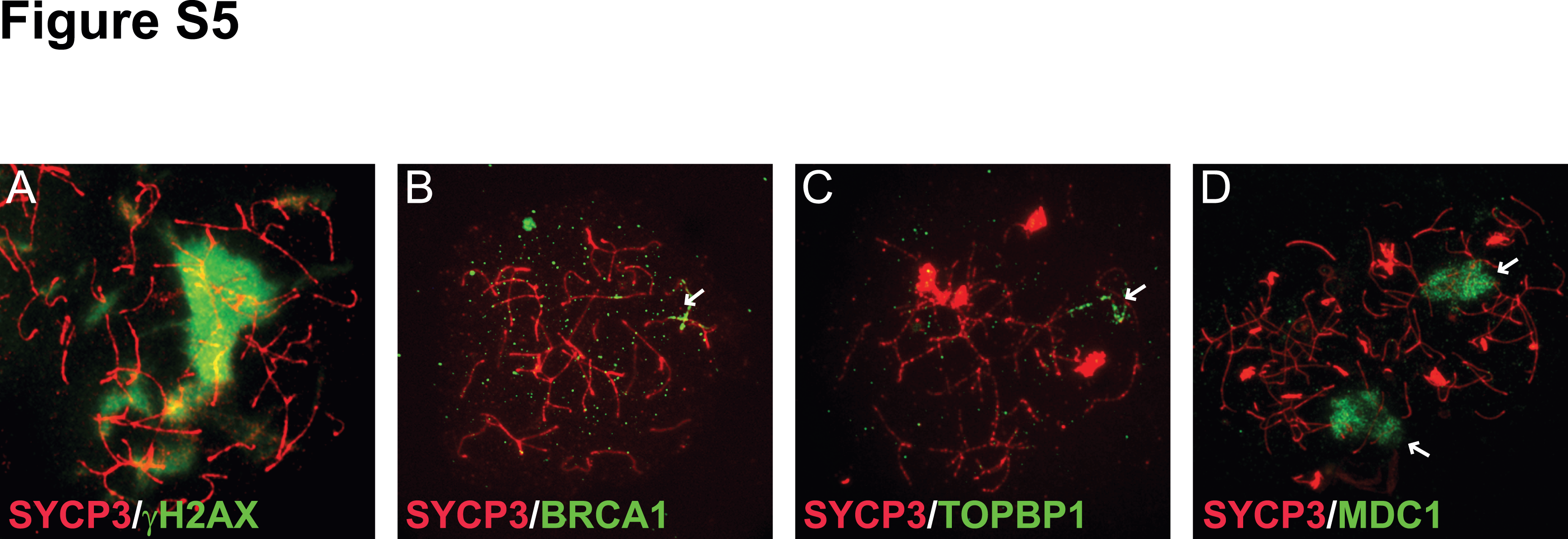

Supplement: Figure S5 — Pseudo XY body in Spo11YF/YF spermatocytes. (A–D) Double immunostaining of Spo11YF/YF spermatocytes with anti-SYCP3 (red) and different DNA repair proteins or histone modifications (green). Antibodies used for immunostaining are indicated. Arrows mark the localization of the pseudo XY body. (TIF) [file pgen.1003538.s005.tif]

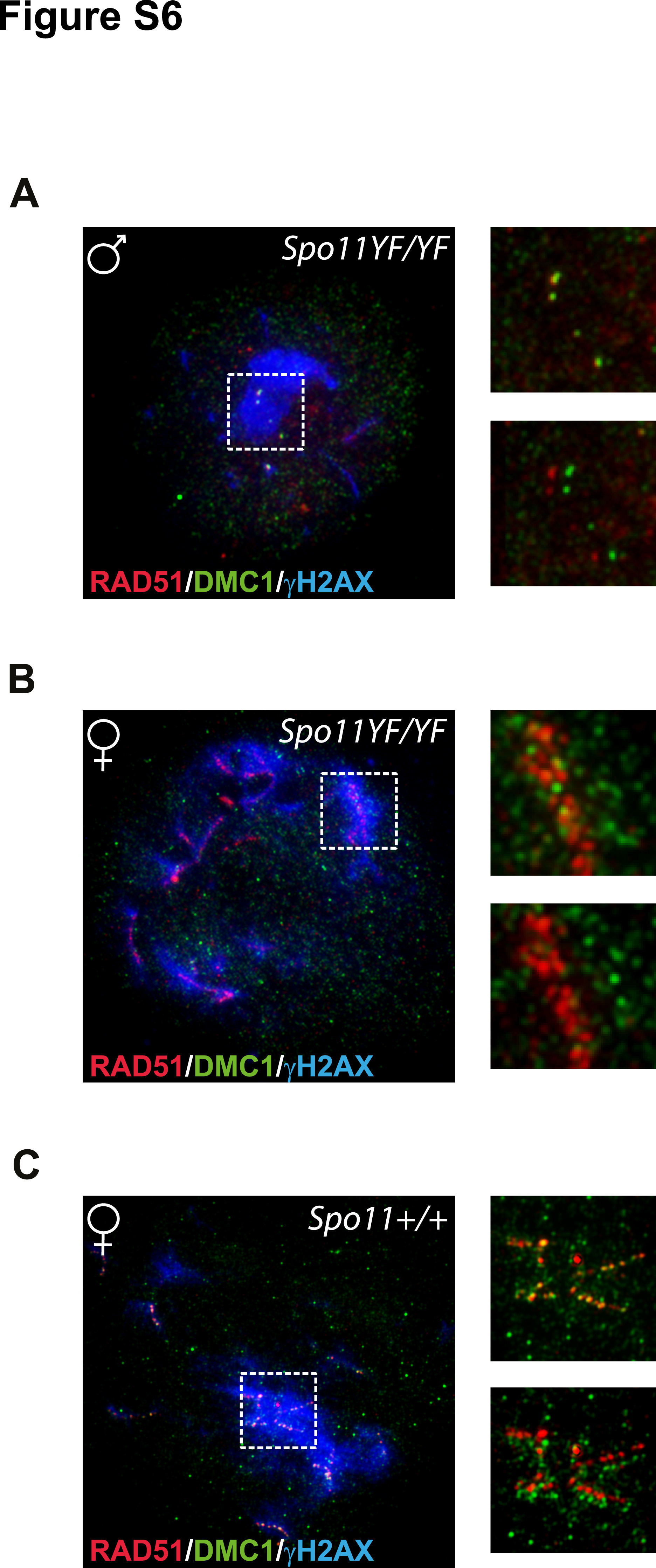

Supplement: Figure S6 — RAD51 and DMC1 foci colocalize in mouse meiocytes. (A–C) Immunostaining of Spo11YF/YF spermatocyte (A), Spo11YF/YF E17.5 oocyte (B), and Spo11+/+ E17.5 oocyte (C) nuclei with anti-RAD51 (red), anti-DMC1 (green) and anti-γH2AX (blue). Close-ups show RAD51 and DMC1 foci in the area of the pseudo XY body next to every nucleus: red and green channels overlaid (top) and offset (bottom). (TIF) [file pgen.1003538.s006.tif]

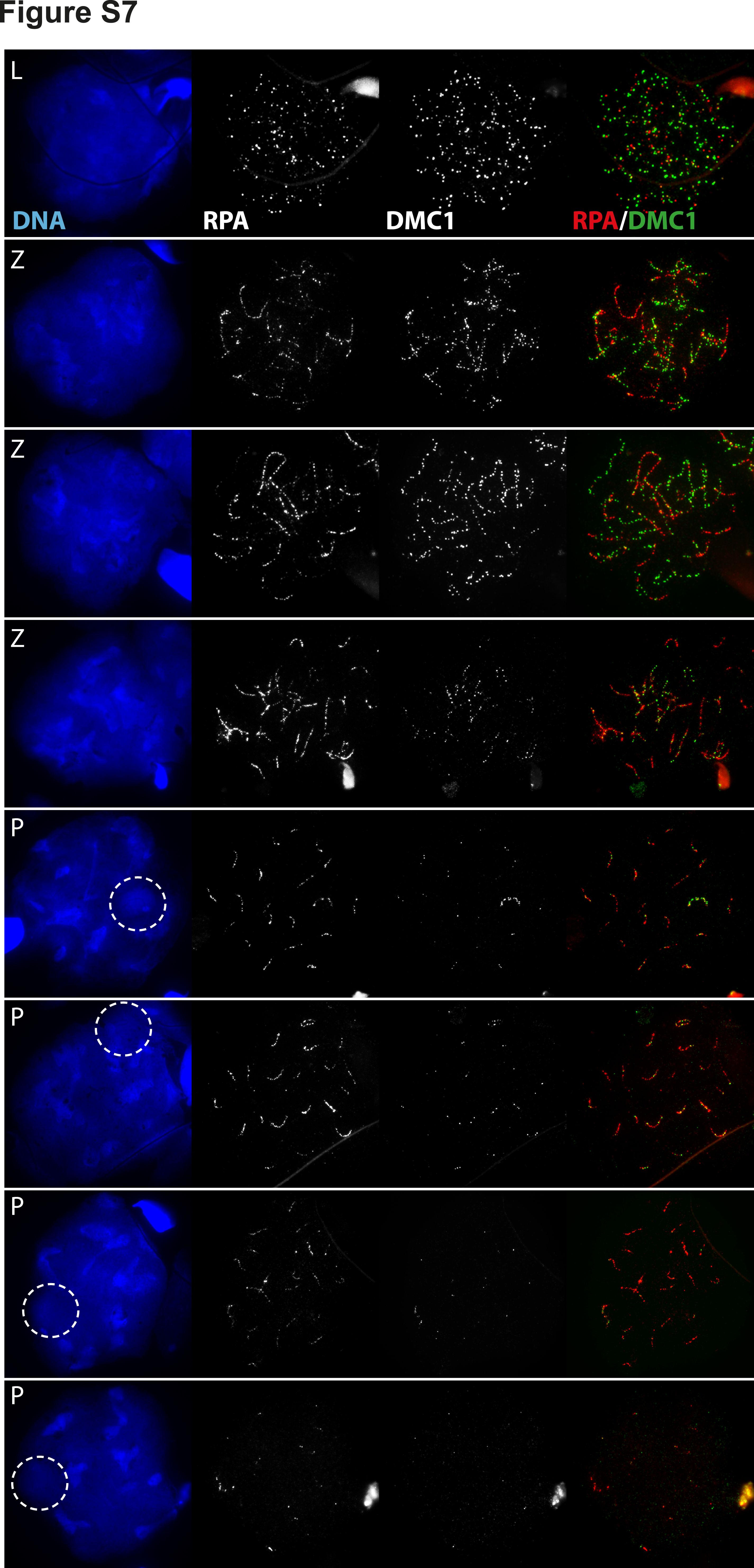

Supplement: Figure S7 — Limited colocalization of RPA and DMC1 during spermatogenesis. Mouse spermatocyte nuclei were stained with anti-DMC1 (green) and anti-RPA (red). DAPI was used to visualize the DNA and stage spermatocytes from leptotene (L) through zygotene (Z) to pachytene (P). Early to late pachytene spermatocytes were distinguished based on the conformation of the X and Y chromosomal axes, that were visible in the DAPI image. Consecutive prophase stages are shown from top to bottom. Dashed circles show the nuclear area of the sex body. Both RPA and DMC1 are very abundant at the onset of meiosis. Most likely, RPA is first loaded on the processed 3′ ssDNA strands, and then replaced by DMC1 and RAD51. Starting from late zygotene onwards, DMC1 foci decrease in number, presumably because the recombinase has accomplished its function and its presence is no longer needed. At the same time RPA is recruited again to protect areas of ssDNA generated during the recombination process. Note that at early pachytene, the X chromosome is clearly enriched for DMC1 but not for RPA foci. However, RPA foci increase on the X at late pachytene, when almost all DMC1 and autosomal RPA foci have disappeared. In general, colocalization of DMC1 and RPA is only sporadically observed at all stages examined. (TIF) [file pgen.1003538.s007.tif]

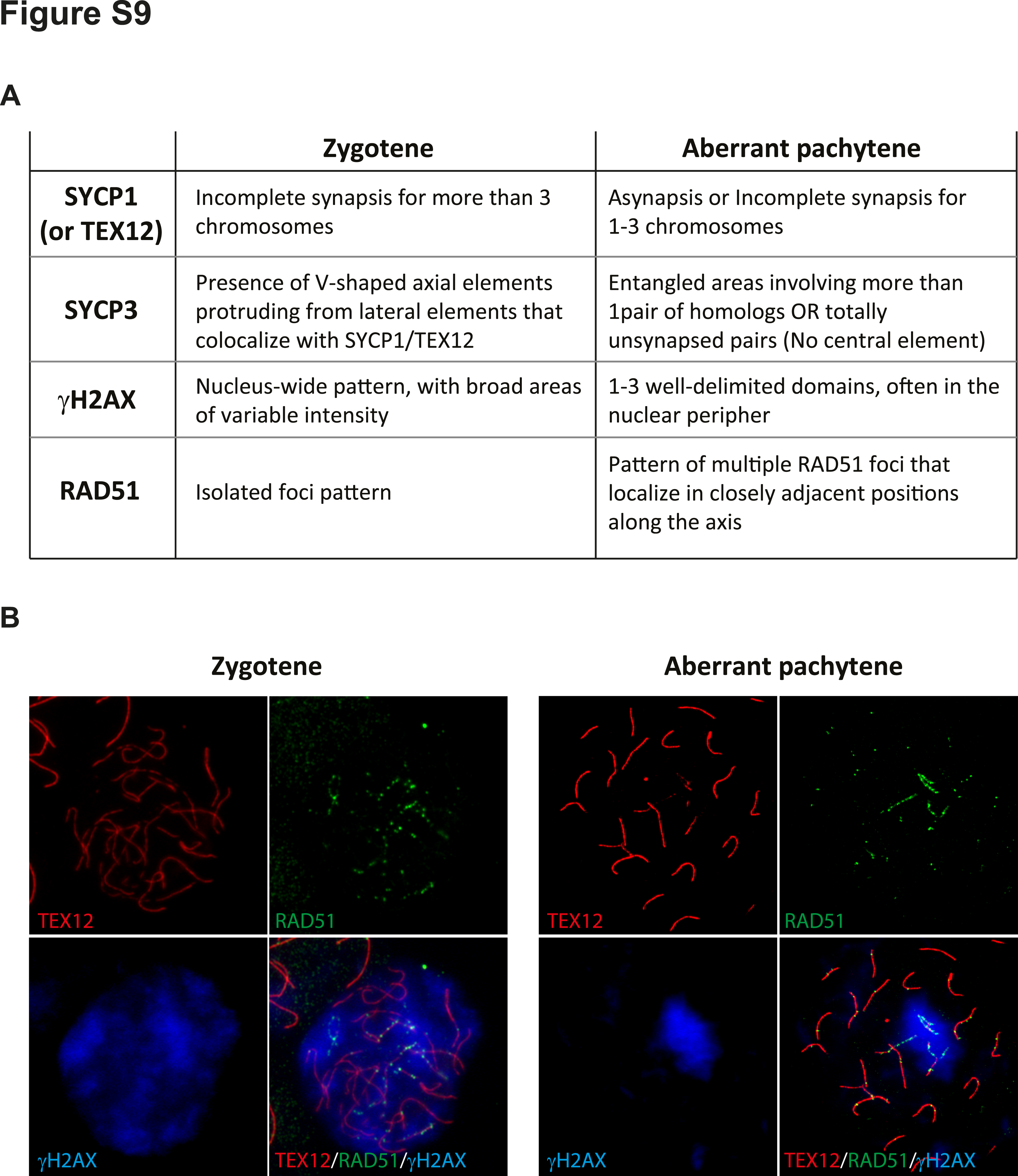

Supplement: Figure S9 — Parameters to discriminate between zygotene and aberrant pachytene wild type oocytes. (A) Summary of the applied parameters to discriminate between zygotene oocytes and aberrant pachytene oocytes. Patterns of SYCP3, SYCP1 (or TEX12), γH2AX, and RAD51 are described for both categories. (B) Representative images of the described oocyte categories. Oocytes were immunostained for TEX12 (red), RAD51 (green), and γH2AX (blue). (TIF) [file pgen.1003538.s009.tif]
